# Unconventional tonicity-regulated nuclear trafficking of NFAT5 mediated by KPNB1, XPOT and RUVBL2

**DOI:** 10.1242/jcs.259280

**Published:** 2022-07-12

**Authors:** Chris Y. Cheung, Ting-Ting Huang, Ning Chow, Shuqi Zhang, Yanxiang Zhao, Mary P. Chau, Wing Cheung Chan, Catherine C. L. Wong, Daniela Boassa, Sebastien Phan, Mark H. Ellisman, John R. Yates, SongXiao Xu, Zicheng Yu, Yajing Zhang, Rui Zhang, Ling Ling Ng, Ben C. B. Ko

**Affiliations:** 1 Department of Applied Biology and Chemical Technology, The Hong Kong Polytechnic University, Hong Kong, China; 2 State Key Laboratory of Chemical Biology and Drug Discovery, The Hong Kong Polytechnic University, Hong Kong, China; 3 Center for Precision Medicine Multi-Omics Research, Health Science Center, Peking University, China Clinical Laboratory Department, The Cancer Hospital of the University of Chinese Academy of Sciences, 102206, Beijing, China; 4 Department of Neurosciences, University of California, San Diego, La Jolla, CA 92093, USA; 5 Center for Research in Biological Systems, National Center for Microscopy and Imaging Research, University of California, San Diego, La Jolla, CA 92093, USA; 6 Department of Chemical Physiology, The Scripps Research Institute, La Jolla, CA 92037, USA; 7 The Clinical Laboratory Department, The Cancer Hospital of the University of Chinese Academy of Sciences (Zhejiang Cancer Hospital), Institute of Basic Medicine and Cancer, Chinese Academy of Sciences, Hangzhou, 310000, Zhejiang, China

**Keywords:** NFAT5, RUVBL2, Nucleocytoplasmic trafficking, Importin, Exportin-T

## Abstract

NFAT5 is the only known mammalian tonicity-responsive transcription factor with an essential role in cellular adaptation to hypertonic stress. It is also implicated in diverse physiological and pathological processes. NFAT5 activity is tightly regulated by extracellular tonicity, but the underlying mechanisms remain elusive. Here, we demonstrate that NFAT5 enters the nucleus via the nuclear pore complex. We found that NFAT5 utilizes a unique nuclear localization signal (NFAT5-NLS) for nuclear import. siRNA screening revealed that only karyopherin β1 (KPNB1), but not karyopherin α, is responsible for the nuclear import of NFAT5 via direct interaction with the NFAT5-NLS. Proteomics analysis and siRNA screening further revealed that nuclear export of NFAT5 under hypotonicity is driven by exportin-T (XPOT), where the process requires RuvB-like AAA-type ATPase 2 (RUVBL2) as an indispensable chaperone. Our findings have identified an unconventional tonicity-dependent nucleocytoplasmic trafficking pathway for NFAT5 that represents a critical step in orchestrating rapid cellular adaptation to change in extracellular tonicity. These findings offer an opportunity for the development of novel NFAT5 targeting strategies that are potentially useful for the treatment of diseases associated with NFAT5 dysregulation.

## Abstract (precis)

**Summary:** Tonicity-dependent NFAT5 nucleocytoplasmic trafficking is regulated by an importin-β- and exportin-T-regulated mechanism.

## INTRODUCTION

The nuclear envelope consists of two lipid bilayer membranes that compartmentalize the nucleus from the cytosol. It is perforated with nuclear pore complexes (NPCs), which are composed of proteins known as nucleoporins (Nups). NPCs serve as a gateway to actively control nucleocytoplasmic exchange of macromolecules such as proteins and RNA (D'Angelo and Hetzer, 2008). The control of nuclear availability of these substances, including transcription factors, by nucleocytoplasmic trafficking through NPCs is one of the key mechanisms for regulating gene transcription programs. Under most circumstances, the passage of transcription factors across NPCs requires the assistance of specific nucleocytoplasmic transport receptors (NTRs) belonging to the karyopherin (Kap) family (Wing et al., 2022). Members of the Kap family can be further subdivided into subgroups according to their role in moving cargo into the nucleus (importins), out of the nucleus (exportins), or in both directions (biportins). In the human genome, ten importins, five exportins and three biportins have been identified. These receptors recognize specific linear nuclear targeting elements known as nuclear localization signals (NLSs), which promote nuclear import, or nuclear export signals (NESs), or specific folded domains in the cargo (Wing et al., 2022). Among them, the oldest characterized nuclear targeting elements are known as the classical nuclear localization signal (cNLS) and the classical NES. Subsequently, several different classes of NLSs, which are characterized by different amino acid compositions and lengths, have been identified (Wing et al., 2022).

Nuclear factor of activated T-cells 5 (NFAT5), also known as the osmotic response element-binding protein (OREBP) or tonicity-responsive element-binding protein (TonEBP), is a Rel homology domain-containing protein that belongs to the NFAT family. It is also the only known tonicity-dependent transcription factor in mammals (López-Rodríguez et al., 1999; Ko et al., 2000; Miyakawa et al., 1999). NFAT5 is indispensable for cell survival in a hypertonic milieu, where it restores cellular homeostasis by orchestrating a genetic program that promotes the expression of heat shock proteins and replaces detrimental cellular electrolytes with cell-compatible organic osmolytes (Burg et al., 2007). The kidney inner medulla was once considered the only physiological relevant site for the activation and activity of NFAT5. Nevertheless, recent findings have suggested the presence of hypertonic stress in certain tissue microenvironments (Go et al., 2004; Machnik et al., 2009), and NFAT5 activation has been associated with a plethora of physiological and pathological processes, including blood pressure regulation, inflammation and development of autoimmune diseases (Machnik et al., 2009; Kleinewietfeld et al., 2013).

NFAT5 interacts with its cognate enhancer, known as osmotic response element (ORE) or tonicity responsive element (TonE) (Takenaka et al., 1994; Ko et al., 1997), for gene transcriptions (Ko et al., 2000; Ferraris et al., 2002). Its activity is controlled by intricate, yet elusive, mechanisms. Nuclear availability plays an important role in the regulation of NFAT5-dependent gene transcription. Under isotonic conditions, NFAT5 is localized pan-cellularly as a result of an equilibrium between continuous nuclear import and export mediated by a putative cNLS and an NES, respectively (Tong et al., 2006). Upon hypertonic induction, NFAT5 predominantly accumulates in the nucleus and upregulates NFAT5-dependent gene transcriptions. Conversely, hypotonicity inactivates NFAT5 by promoting its nuclear export in a NES-independent manner (Tong et al., 2006). A novel NES, which is known as the auxiliary export domain (AED) and does not bear sequence homology to any other known sequences, is crucial for hypotonicity-induced NFAT5 nuclear export (Xu et al., 2008). The corresponding NTRs involved in nuclear import and hypotonicity-mediated nuclear export of NFAT5 have remained elusive. More importantly, how these domains work in a concerted manner to fine-tune the nuclear abundance of NFAT5 in response to changes in tonicity remains unknown.

To understand how NFAT5 activity is regulated by tonicity, we sought to delineate the underlying mechanisms of NFAT5 nucleocytoplasmic trafficking. We found that NFAT5 nuclear import is mediated by a previously uncharacterized NLS that relies on karyopherin β1 (KPNB1, also known as importin β1) as the only NTR for import. We also discovered that hypotonicity-induced nuclear export of NFAT5 requires exportin-T (XPOT) as the NTR and RUVBL2 as an indispensable chaperone. Our findings have shed light on the unique nuclear import pathway for cellular adaptation to changes in tonicity and have opened up an opportunity for targeting NFAT5 activity in order to modulate diverse cellular processes.

## RESULTS

### An extensive NLS is required for nuclear import of NFAT5

**Fig. 1.**
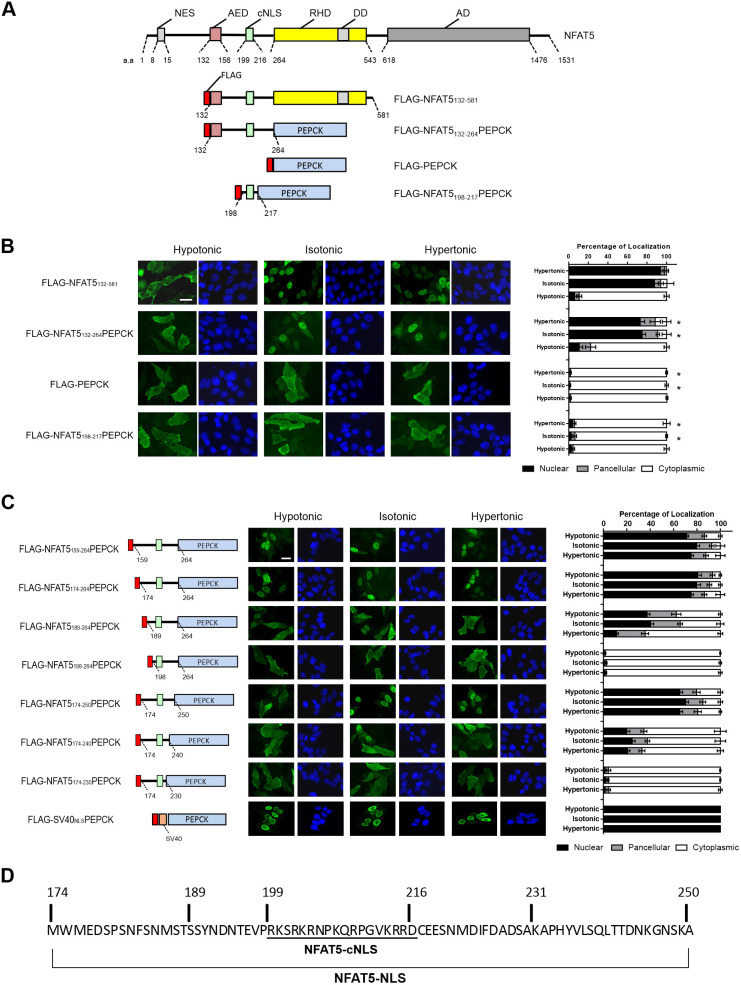
**Mapping of minimal essential nuclear import sequence for NFAT5 nuclear import.** (A) Schematic illustration of NFAT5, FLAG–NFAT5_132–581_ and FLAG–NFAT5–PEPCK fusion constructs. DD, dimerization domain; AD, putative transcriptional activation domains; a.a., NFAT5 amino acid residue numbers. (B) Left: representative cellular immunofluorescence images of the indicated FLAG fusion proteins in HeLa cells treated with hypotonic, isotonic or hypertonic medium for 90 min. Cells were stained with FLAG antibody and FITC-labeled secondary antibody (green, left-hand images), and were counterstained with DAPI (blue, right-hand images). Scale bar: 30 µm. Right, quantitative analysis of subcellular localization of fluorescence signal of FLAG–NFAT5 and FLAG–NFAT5–PEPCK constructs in each condition. (C) Mapping of the minimal essential NLS using the indicated FLAG–NFAT5–PEPCK deletion mutant constructs. Left: schematic illustration of the mutant constructs, color-coded as in A. Amino acids numbers are shown based on the full-length NFAT5 sequence. Middle: representative immunofluorescence images of cells expressing the recombinant FLAG fusion constructs. Cells were treated with hypotonic, isotonic or hypertonic medium for 90 min. After fixation, cells were stained with FLAG antibody and FITC-labeled secondary antibody (green, left-hand images), and were counterstained with DAPI (blue, right-hand images). Scale bar: 30 µm. Right: quantitative analysis of subcellular localization of the FLAG fusion protein fluorescence signal in HeLa cells expressing the indicated fusion constructs in each condition. In B and C, at least 100 cells were scored in each condition. Data are presented as mean±s.e.m. of three independent experiments. *P<0.0001 (one-way ANOVA with Bonferroni's multiple comparison test). (D) Amino acid sequence of the NFAT5-NLS required for NFAT5 nuclear import, with location of the cNLS indicated.

Our previous studies have shown that FLAG–OREBP_1–581_Δ1–131 (renamed hereafter as FLAG–NFAT5_132–581_; Fig. 1A) (Xu et al., 2008; Tong et al., 2006), a truncated form of NFAT5 devoid of both the N-terminal NES and C-terminal transactivation domain, faithfully recapitulates nucleocytoplasmic trafficking properties of endogenous NFAT5 in response to changes in extracellular tonicity. This truncated recombinant NFAT5 contains an AED (amino acids 132–156), which is indispensable for nuclear export under hypotonicity; a putative cNLS (amino acids 199–216, referred to here as NFAT5-cNLS), as suggested by analysis using Motif Scan (http://myhits.isb-sib.ch/cgi-bin/motif_scan); and a Rel homology DNA-binding domain (RHD; amino acids 264–581) (Tong et al., 2006; Xu et al., 2008). However, it does not contain the NES (amino acids 8–15) that is responsible for nucleocytoplasmic shuttling of NFAT5 under isotonic conditions (Tong et al., 2006; Cheung and Ko, 2013). Therefore, FLAG–NFAT5_132–581_ is constitutively localized to the nucleus under isotonic conditions, and the AED drives nuclear export activity of this recombinant protein under hypotonicity (Tong et al., 2006). We generated deletion mutants to decipher the minimal protein domains required for NFAT5 nucleocytoplasmic trafficking (Fig. 1A). First, we elucidated whether the RHD is dispensable by replacing it with cytoplasmic phosphoenolpyruvate carboxykinase (PEPCK-C, also known as PCK1). PEPCK-C is an exclusively cytoplasmic protein used as a reporter for the characterization of NLS activity (Theodore et al., 2008). The apparent molecular mass of this new fusion protein (FLAG–NFAT5_132–264_PEPCK) is 83 kDa (Fig. S1A), which precludes a passive nuclear transport mechanism. Both FLAG–NFAT5_132–581_ and FLAG–NFAT5_132–264_PEPCK predominantly localized to the cytoplasm and the nucleus when cells were subjected to hypotonic and hypertonic challenge, respectively (Fig. 1B). Replacement of the RHD with PEPCK-C resulted in a modest reduction in nuclear localization of the recombinant protein under both isotonic and hypertonic conditions, suggesting that the RHD has a minimal impact on NFAT5 nuclear import. However, a fusion protein consisting of the putative NFAT5-cNLS and PEPCK-C (FLAG–NFAT5_198–217_PEPCK) (Fig. 1A) failed to enter the nucleus regardless of extracellular tonicity (Fig. 1B). As a control, FLAG-tagged PEPCK-C (FLAG–PEPCK) was observed to exclusively localize to the cytoplasm under all tonicities examined. Our findings suggest that amino acid residues 132–264 of NFAT5 confer tonicity-sensitive nuclear import and export activity to a heterologous protein, whereas the NFAT5-cNLS per se is an inactive nuclear import signal. Nevertheless, alanine substitution of the three core residues (_202_RKR_204_) in NFAT5-cNLS has been shown to result in complete ablation of nuclear import of NFAT5 (Tong et al., 2006), suggesting that NFAT5-cNLS is essential but insufficient to confer significant nuclear import activity. To further delineate the minimal essential sequence required for nuclear import activity, we conducted nested deletion of FLAG–NFAT5_132–264_PEPCK from the N- and C-terminal ends of the NFAT5 fragment and determined nucleocytoplasmic localization of the truncated proteins under different tonicities. As shown in Fig. 1C, consistent with our previous findings that the AED is important for nuclear export (Tong et al., 2006), AED-deficient fusion protein (FLAG–NFAT5_159–264_PEPCK) became constitutively localized to the nucleus under different tonicities. Deletion mapping analysis suggested that the presence of amino acid residues 159–173 of NFAT5 (which were removed in FLAG–NFAT5_174–264_PEPCK) is not required for nuclear trafficking of the fusion protein. Nevertheless, nuclear localization of the fusion protein was profoundly inhibited when amino acid residues 174–188 of NFAT5 were removed from the fusion protein (FLAG–NFAT5_189–264_PEPCK), and was completely abolished when amino acid residues 189–197 of NFAT5 were deleted (FLAG–NFAT5_198–264_PEPCK). On the other hand, amino acid residues 251–264 of NFAT5 (FLAG–NFAT5_174–250_PEPCK) were not required for nuclear localization of the fusion protein, but deletion of NFAT5 amino acid residues 241–250 from the fusion protein (FLAG–NFAT5_174–240_PEPCK) impaired nuclear localization significantly. Furthermore, additional deletion of NFAT5 amino acid residues 231–240 (FLAG–NFAT5_174-–230_PEPCK) resulted in exclusive cytoplasmic localization of the fusion protein. As a control, a fusion protein containing a cNLS from SV40 (SV40_NLS_) and PEPCK-C was used, and this fusion protein was observed to constitutively localize to the nucleus. Taken together, these data suggest that the presumed NFAT5-cNLS (Fig. 1D) does not serve as a functional NLS. A minimal protein domain comprised of 52 amino acids (residues 189–240) is required for directing nuclear import of NFAT5. However, a domain comprised of 77 amino acids (residues 174–250) exhibits full nuclear import activity (Fig. 1D). We named this NLS the NFAT5-NLS (Fig. 1D).

### Nucleocytoplasmic trafficking of NFAT5 is mediated through the nuclear pore

**Fig. 2.**
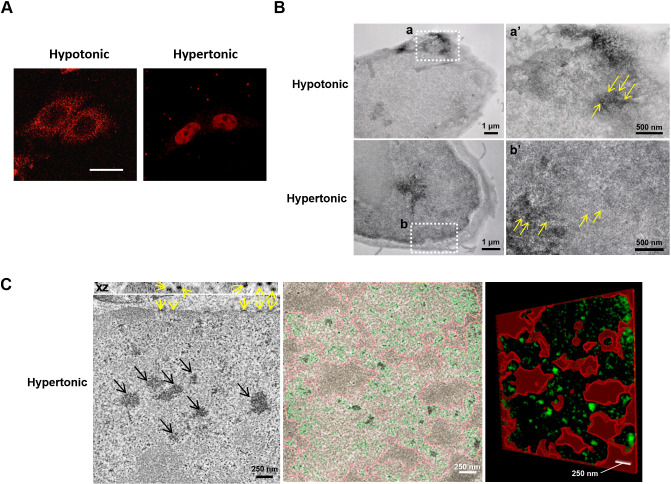
**Correlated light and electron microscopy analysis of NFAT5.** (A) HeLa cells transfected with NFAT5_174–250_–MiniSOG. Fluorescence signals (red) were visualized in the indicated conditions using confocal microscopy (63× objective magnification). Scale bar: 30 μm. (B) Spatial distribution of NFAT5_174–250_–MiniSOG following incubation in either hypotonic or hypertonic conditions. Following photo-induced oxidation of DAB, NFAT5_174–250_–MiniSOG-expressing HeLa cells were processed and visualized using EM. Under hypotonic conditions, the DAB-positive staining was clearly observed in the cytoplasm. The white square (a) corresponds to the region shown in the high magnification image (a′). Yellow arrows in a′ indicate staining associated with nuclear pores. In contrast, under hypertonic conditions, the intense DAB-positive staining was observed in the nucleus. The white square (b) corresponds to the region shown in the high magnification image (b′). Yellow arrows in b′ indicate staining associated with nuclear pores. (C) 3D electron tomographic analysis of hypertonicity-treated HeLa cells expressing NFAT5_174–250_–MiniSOG revealed a pattern of staining within the nucleus exclusively associated with regions of less condensed chromatin (black arrows, left image). Yellow arrows indicate staining at the nuclear pores, which is particularly visible in the *x*–*z* plane (top). Segmentation of a tomogram based on thresholding highlights the NFAT5 staining (in green) exclusively outside of the areas of more condensed chromatin (delimited in red, middle and right image). Images in A–C are representative of three independent experiments.

To visualize nucleocytoplasmic trafficking of NFAT5 in response to changes in extracellular tonicity *in situ*, we conducted correlated light and electron microscopy (CLEM) analysis (Ellisman et al., 2012). CLEM takes advantage of the mini singlet oxygen generator (MiniSOG), a small (106 amino acids) fluorescent flavoprotein that generates reactive oxygen species upon exposure to blue light (Ludwig et al., 2013). Local production of reactive oxygen species in glutaraldehyde-stabilized cells then leads to photooxidation of diaminobenzidine (DAB) into an osmiophilic electron-dense polymer, which allows proteins to be visualized at high spatial resolution and contrast under electron microscopy (EM), with good preservation of ultrastructures (Ludwig et al., 2013; Ou et al., 2012). We generated an NFAT5–MiniSOG fusion gene (NFAT5_174–250_–MiniSOG). The fusion protein, which contains both the AED and NFAT5-NLS, was subjected to cytoplasmic and nuclear localization treatment with hypotonicity and hypertonicity, respectively (Fig. 2A). Photooxidation of glutaraldehyde-fixed NFAT5_174–250_–MiniSOG-expressing cells in the presence of DAB resulted in differential deposition of brown reaction products. Electron micrographs of plastic-embedded and osmium-stained sections revealed that electron-dense staining was primary localized to the cytoplasm and nucleus in response to hypotonic and hypertonic treatment, respectively (Fig. 2B). Under hypotonic conditions, ring-shaped staining at the nuclear rim was observed at higher magnifications, consistent with the structure of nuclear pores (yellow arrows in Fig. 2B, panel a′), suggesting that the fusion proteins were captured at the nuclear pore during nuclear export. Under hypertonic conditions, ring-shaped staining at the nuclear rim consistent with the structure of nuclear pores was also observed (yellow arrows in Fig. 2B, panel b′). Interestingly, imaging by electron tomography revealed a DAB-positive patchy staining within the nucleus, which distributed exclusively in regions outside of the most condensed chromatin (black arrows in Fig. 2C), consistent with the notion that active transcription occurs at the fringes of chromosome territories (van Steensel and Furlong, 2019). Moreover, the patchy signals were of different intensities, which might represent clustering of the reporter at different genomic locations. Three-dimensional (3D) electron tomographic analysis of hypertonicity-treated cells further confirmed increased staining density at the nuclear pore (yellow arrows in Fig. 2C). Taken together, these data provide direct evidence that NFAT5 distributes in the nucleus in areas with less condensed chromatin and undergoes nucleocytoplasmic trafficking through the NPC.

### KPNB1 is the nuclear import receptor for NFAT5

**Fig. 3.**
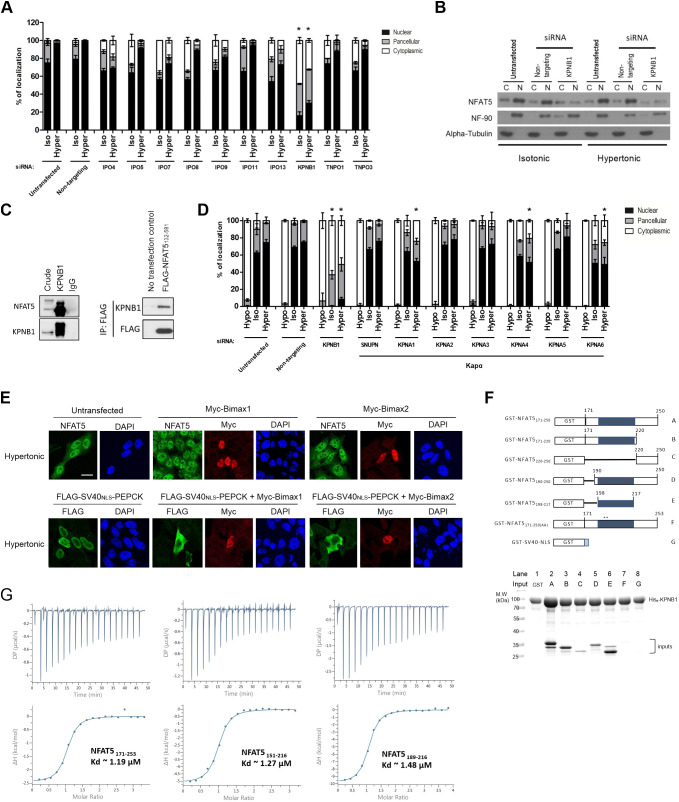
**Identification of nuclear import receptor for NFAT5.** (A) Quantitative analysis of subcellular localization of FLAG fusion protein fluorescence signal in HeLa cells transfected with FLAG–NFAT5_132–264_PEPCK and siRNA targeting the indicated members of the Kapβ family, followed by treatment with isotonic (Iso) or hypertonic (Hyper) medium for 90 min. Cells were stained with FLAG antibody and FITC-labeled secondary antibody, then counterstained with DAPI. At least 100 cells were scored in each condition. Data are presented as mean±s.e.m. of three independent experiments. (B) Western blotting analysis showing cytoplasmic (C) and nuclear (N) distribution of endogenous NFAT5 in HeLa cells transfected with siRNA targeting KPNB1 under different extracellular tonicities. NF-90 and α-tubulin antibodies were used as nuclear and cytoplasmic markers, respectively. Blots are representative of three independent experiments. (C) Left: co-immunoprecipitation analysis of KPNB1 and NFAT5. Immunoprecipitation was carried out using anti-KPNB1 antibodies, and immunocomplexes were subjected to western blot analysis using the NFAT5 and KPNB1 antibodies. Crude input lysate (5% total lysate) and IgG-only samples are shown as controls. Right: co-immunoprecipitation analysis of FLAG–NFAT5_132–581_ and KPNB1. Immunoprecipitation (IP) was carried out using anti-FLAG affinity resin, and immunocomplexes were subjected to western blot analysis using the KPNB1 and FLAG antibodies. Blots shown are representative of three independent experiments. (D) Quantitative analysis of subcellular localization of immunofluorescence signal in HeLa cells expressing FLAG–NFAT5_132–581_ and siRNA targeting the indicated members of the Kapα family or KPNB1. Cells were switched to hypotonic (Hypo), isotonic or hypertonic medium for 90 min before fixation, followed by staining with FLAG antibody and FITC-labeled secondary antibody, and counterstaining with DAPI. At least 100 cells were scored in each condition. Data are presented as mean±s.e.m. of three independent experiments. (E) Representative cellular immunofluorescence images of NFAT5 and FLAG–SV40_NLS_–PEPCK in HeLa cells expressing Myc–Bimax1 or Myc–Bimax2. HeLa cells treated with hypertonic medium for 30 min were co-stained with antibodies against NFAT5 and Myc, or against FLAG and Myc, followed by staining with FITC- and TRITC-labeled secondary antibodies. Nuclei were counterstained using DAPI. Scale bar: 30 μm. Images are representative of three independent experiments. (F) Top: schematic representation of different GST–NFAT5 and GST­–SV40_NLS_ fusion proteins. Shaded boxes represent the NFAT5-NLS and numbers indicate NFAT5 amino acid positions. Asterisks mark the position of the mutated residues in GST–NFAT5_171–250(AA)_. Bottom: *in vitro* pulldown analysis using His–KPNB1. For lanes 1–8, His–KPNB1 was immobilized on Ni-NTA agarose, and the indicated GST–NFAT5 fusion proteins (as labeled in the schematic) or GST alone were added. After washing, proteins were eluted with excess imidazole. Proteins were analyzed using SDS–PAGE followed by Coomassie Blue staining. Gel shown is representative of three independent experiments. (G) ITC profiles to measure the binding affinity of NFAT5 to KPNB1. Different constructs of NFAT5 putative NLS region, including residues 171–253, 151–216 and 189–216, showed similar binding affinities (*Kd*). DP, differential power; ΔH, enthalpy. Data shown are representative of three independent experiments. In A and D, *P<0.0001 represents significant reduction in nuclear signal compared with that in untransfected cells under the same tonicity (one-way ANOVA with Bonferroni's multiple comparison test).

We sought to determine the NTR responsible for directing NFAT5 nuclear import. To systematically evaluate the involvement of karyopherin β (Kapβ) in this process, we conducted siRNA knockdown to deplete Kapβ proteins that are known to mediate nuclear import via recognizing distinct classes of NLS on import cargoes (Soniat and Chook, 2015), followed by determination of subcellular localization of FLAG–NFAT5_132–264_PEPCK. Among others, only depletion of KPNB1 – but not of TNPO1 (Kapβ2), IPO4 (importin 4), IPO5 (importin 5), IPO7 (importin 7), IPO8 (importin 8), IPO9 (importin 9), IPO11 (importin 11), IPO13 (importin 13), or TNPO3 (Trn-SR) – markedly inhibited nuclear localization of FLAG–NFAT5_132–264_PEPCK under both isotonic and hypertonic conditions (Fig. 3A; Fig. S1B,C). Nuclear abundance of NFAT5 was diminished significantly in KPNB1-depleted cells (Fig. 3B). Co-immunoprecipitation of KPNB1 resulted in the presence of endogenous NFAT5 in the immunocomplex (Fig. 3C, left), and co-immunoprecipitation of FLAG–NFAT5_132–581_ resulted in the presence of endogenous KPNB1 in the immunocomplex (Fig. 3C, right). Taken together, these data suggest that KPNB1 is associated with NFAT5. KPNB1 is known to heterodimerize with nuclear import adaptor karyopherin α (Kapα) for nuclear transport of cNLS-containing cargo (Marfori et al., 2011). siRNA knockdown was used to determine whether any members of the Kapα family – including KPNA1 (IPOA5), KPNA2 (IPOA1), KPNA3 (IPOA4), KPNA4 (IPOA3), KPNA5 (IPOA6), KPNA6 (IPOA7), and KPNA7 – or SNUPN (snurportin 1), a novel nuclear import adaptor of KPNB1 (Huber et al., 1998), are involved in the nuclear import of NFAT5. Nuclear localization of FLAG–NFAT5_132–264_PEPCK was not significantly altered by the knockdown of any of these Kapα members (Fig. 3D), suggesting that KPNB1 mediates nuclear import of NFAT5 in the absence of nuclear import adaptors from the Kapα family. To further confirm that Kapα is not involved in NFAT5 nuclear import, we tested the effect of ectopic expression of Bimax1 or Bimax2, which are potent peptide inhibitors for the Kapα/β pathway (Kosugi et al., 2008), in NFAT5 nuclear import. While ectopic expression of Bimax1 or Bimax2 blocked nuclear import of SV40_NLS_-driven cargo (FLAG–SV40_NLS_–PEPCK), nuclear import of NFAT5 was not affected (Fig. 3E). These data unequivocally confirmed that Kapα is not involved in NFAT5 nuclear import.

We conducted an *in vitro* pulldown assay to test whether recombinant NFAT5-NLS proteins (Fig. 3F; Fig. S1D) and KPNB1 interact directly. Agarose beads coupled to His–KPNB1 efficiently pulled down GST–NFAT5_171–250_, but not GST control (Fig. 3F, compare lane 2 and lane 1). Truncation of GST–NFAT5_171–250_ from either terminus moderately weakened its interaction with KPNB1 (Fig. 3F, compare lanes 3 and 5 with lane 2). Intriguingly, despite the failure of NFAT5-cNLS to confer nuclear import activity to PEPCK, GST–NFAT5_198–217_, which contains NFAT5-cNLS only, interacts significantly with His–KPNB1 at a level comparable to that of GST–NFAT5_171–250_ (Fig. 3F, compare lane 6 and lane 2). However, their interaction was nearly abolished when the NFAT5-cNLS (amino acids 199–216) was removed (Fig. 3F, lane 4), or when the core basic amino acids (R202 and K203) were mutated to alanine (GST–NFAT5_171–250(AA)_; Fig. 3F, lane 7), a result that was consistent with the notion that these residues are important for nuclear import activity. Taken together, these data confirm direct interaction between NFAT5-NLS and KPNB1, with the central NFAT5-cNLS fragment most critical, while the N- and C-terminal regions serve a supportive role. To further understand this interaction, we conducted isothermal titration calorimetry (ITC) experiments to measure the binding affinity of different NFAT5 fragments to full-length KPNB1. We found that NFAT5_171–253_ and NFAT5_151–216_ bind to KPNB1 with almost the same binding affinity as NFAT5_189–216_, which only comprises the NFAT5-cNLS (*K_d_* of ∼1.2 μM, ∼1.3 μM and ∼1.5 μM, respectively; Fig. 3G). These ITC measurements corroborated the results of our pulldown assays and further showed that different NLS fragments interact with KPNB1 with similar affinity and that NFAT5-cNLS is most critical for KPNB1 binding.

### *In vitro* reconstitution of KPNB1-mediated nuclear import

As there is a discrepancy between the amino acid residues of NFAT5 required for effective KPNB1 interaction *in vitro* and for effective nuclear import *in vivo*, an *in vitro* nuclear transport assay was conducted using digitonin-permeabilized HeLa cells to further discern the underlying mechanism. Recombinant His-tagged NFAT5-NLS fused to monomeric green fluorescent protein (AcGFP) (His–NFAT5_171–250_–AcGFP), His-tagged SV40 large T antigen NLS fused to AcGFP (His–SV40_NLS_–AcGFP) and His-tagged AcGFP (His–AcGFP) were expressed in bacteria and purified (Fig. S2A), and their nuclear import activities were evaluated. Both His–NFAT5_171–250_–AcGFP and His–SV40_NLS_–AcGFP underwent nuclear import dependent on the presence of cytosolic extracts, ATP-regeneration mixture and RanGTP, whereas the import was abolished in the presence of the nuclear pore inhibitor wheat germ agglutinin (WGA), high concentrations of GTP, or nonhydrolyzable GTP analog (GTPγS) (Fig. S2B). His–AcGFP, which does not contain any NLS, failed to enter the nucleus (Fig. S2B). These findings suggest that, similar to His–SV40_NLS_–AcGFP, His–NFAT5_171–250_–AcGFP enters the nucleus in an ATP- and GTP-dependent manner mediated by cytosolic factors, presumably via the NTRs.

**Fig. 4.**
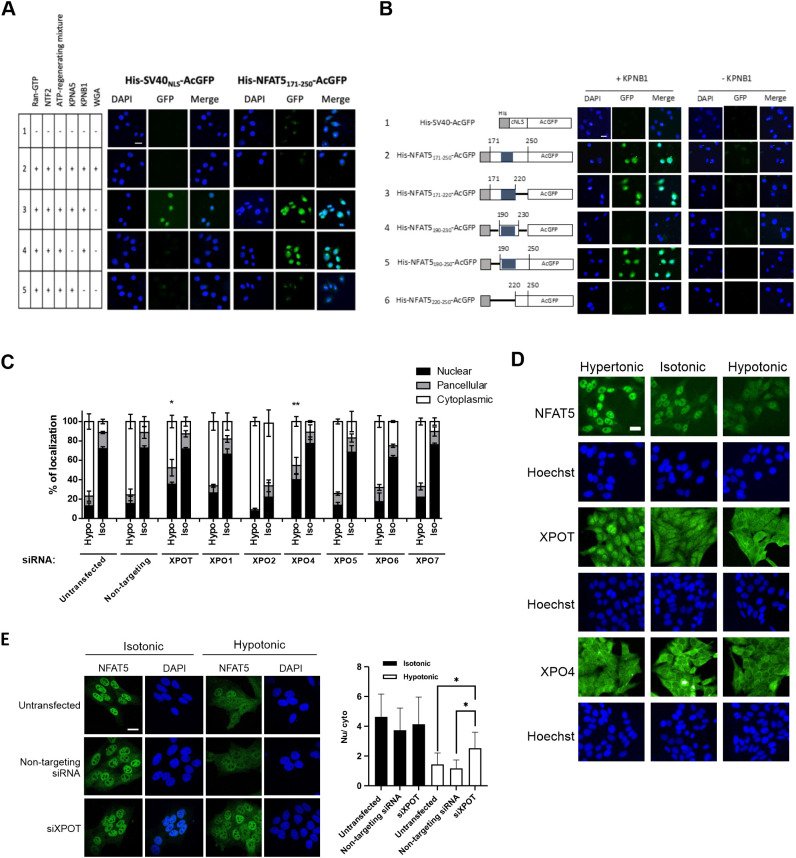
**Identification of importin and exportin for NFAT5 nuclear import and export.** (A) *In vitro* nuclear import assay of His–SV40_NLS_–AcGFP and His–NFAT5_171–250_–AcGFP was carried out using digitonin-permeabilized HeLa cells supplemented with individual nuclear transport factors, as indicated. The transport assay was carried out for 30 min at 37°C. Cells were then fixed with paraformaldehyde and stained with DAPI, and GFP fluorescence images were taken using a confocal microscope. Scale bar: 30 µm. Images are representative of three independent experiments. (B) Role of KPNB1 in nuclear import of His–NFAT5–AcGFP. Left: schematic representation of the indicated His–NFAT5–AcGFP reporter constructs. Shaded regions indicate NFAT5-cNLS. Right: *in vitro* nuclear import assay of His–SV40_NLS_–AcGFP and the indicated His–NFAT5–AcGFP fusions was carried out using digitonin-permeabilized HeLa cells supplemented with RanGTP, NTF2 and ATP-regenerating mixture, in the presence or absence of KPNB1. The transport assay was carried out for 30 min at 37°C. Cells were then fixed with paraformaldehyde and stained with DAPI, and images were taken using a confocal microscope. Scale bar: 30 µm. Images are representative of three independent experiments. (C) Quantitative analysis of subcellular localization of immunofluorescence signal in HeLa cells transfected with FLAG–NFAT5_132–581_ and siRNA targeting the indicated members of the exportin family. Cells were switched to isotonic (Iso) or hypotonic (Hypo) medium for 90 min before fixation. Cells were stained with FLAG antibody and FITC-labeled secondary antibody, and then counterstained with DAPI. For each condition, at least 100 cells were scored. Data are presented as mean±s.e.m. of three independent experiments. *P<0.01 and **P<0.001 indicate significant induction in nuclear fluorescence signal compared with that in the untransfected cells and cells transfected with non-targeting siRNA (one-way ANOVA with Bonferroni's multiple comparison test). (D) Representative immunofluorescence images of subcellular localization of endogenous NFAT5, XPOT and XPO4 in response to treatment with hypotonic, isotonic or hypertonic medium for 90 min. NFAT5, XPOT and XPO4 were stained with the corresponding antibodies. Cells were counterstained with Hoechst. Scale bar: 30 µm. Images are representative of three independent experiments. (E) Left: representative immunofluorescence images of HeLa cells that were either untransfected or transfected with XPOT siRNA (siXPOT) or control non-targeting siRNA. Cells were switched to isotonic or hypotonic medium, as indicated, for 90 min before fixation. Cells were stained with NFAT5 antibody and FITC-labeled secondary antibody, and were counterstained with DAPI. Right: quantitative analysis of nucleocytoplasmic distribution of NFAT5 under different conditions. Nuclear:cytoplasmic fluorescence signal ratio (Nu/cyto) of NFAT5 in cells was calculated by measuring fluorescence intensity in the nucleus and cytoplasm using ImageJ. Data are presented as mean±s.e.m. At least 18 cells in each condition were scored. *P<0.001 (one-way ANOVA with Bonferroni's multiple comparison test). Scale bar: 30 µm.

To further delineate the molecular determinants of His–NFAT5_171–250_–AcGFP nuclear import, we conducted *in vitro* nuclear transport assays with NTRs and defined factors. In the absence of exogenous factors, His–NFAT5_171–250_–AcGFP and His–SV40_NLS_–AcGFP did not undergo nuclear import (Fig. 4A, lane 1). Nuclear translocation of both recombinant proteins was evident in the presence of RanGTP, nuclear transport factor 2 (NTF2), ATP-regenerating mixture, KPNA5 and KPNB1 (Fig. 4A, lane 2), but was abolished by the addition of WGA (Fig. 4A, lane 5). In agreement with the notion that nuclear import of cNLS requires ternary complex formation with importin-α and -β, nuclear accumulation of His–SV40_NLS_–AcGFP was abolished in the absence of KPNA5 (Fig. 4A, lane 4) or KPNB1 (Fig. 3F, lane 5). In contrast, nuclear import of His–NFAT5_171–250_–AcGFP was abolished in the absence of KPNB1 (Fig. 4A, lane 5) but not KPNA5 (Fig. 4A, lane 4). Therefore, the mechanism of NFAT5 nuclear import is mediated by a KPNB1-dependent and Kapα-independent mechanism, which is distinct from the classical SV40 T antigen nuclear import pathway.

We made deletion mutants (Fig. 4B; Fig. S2A) to define the sequence requirement for NFAT5-NLS activity *in vitro*. Compared with import of His–NFAT5_171–250_–AcGFP, neither the deletion of NFAT5 amino acid residues 221–250 (His–NFAT5_171–220_–AcGFP) nor the deletion of NFAT5 amino acid residues 171–189 (His–NFAT5_190–250_–AcGFP) affected KPNB1-mediated nuclear import of the fusion protein (Fig. 4B, lanes 2, 3 and 5). However, deletion of NFAT5 amino acid residues 171–219 (His–NFAT5_220–250_–AcGFP) completely abolished nuclear entry (Fig. 4B, lane 6). A fusion protein containing mainly the cNLS (His–NFAT5_190–230_–AcGFP) failed to undergo nuclear entry (Fig. 4B, lane 4). All fusion proteins only underwent nuclear entry in the presence of KPNB1 (Fig. 4B), suggesting that it is the only nuclear transport receptor required for this process. Interestingly, although the fragment corresponding to NFAT5-cNLS (amino acids 198–217) interacted with KPNB1 *in vitro* (Fig. 3F), both cell-based (Fig. 1C) and *in vitro* reconstitution assays suggested that NFAT5-NLS (amino acids 189–240) is the minimal region required for nuclear import activity of NFAT5.

### Identification of the NTR for NFAT5 nuclear export under hypotonicity

In a previous study we have shown that exportin-1 (XPO1, also known as CRM1) is required for nuclear export of NFAT5 during isotonic nucleocytoplasmic shuttling, acting via the canonical NES (Tong et al., 2006), whereas the AED is required for NFAT5 nuclear export under hypotonicity, although the NTR involved remains unknown (Tong et al., 2006). To identify the putative NTR involved, we conducted siRNA knockdown of exportins, followed by determination of the subcellular localization of FLAG–NFAT5_132–581_ (which does not have an NES, but contains an intact AED) in response to hypotonicity. We successfully knocked down the expression of exportin-T (XPOT), exportin-1 (XPO1), exportin-2 (XPO2), exportin-4 (XPO4), exportin-5 (XPO5), exportin-6 (XPO6) and exportin-7 (XPO7) (Fig. S3A). XPO2 is responsible for nuclear export of KPNA proteins (Solsbacher et al., 1998), but knockdown of XPO2 has been shown to impact protein nuclear import (Dong et al., 2018). Consistent with this notion, gene knockdown of XPO2 resulted in exclusive cytoplasmic localization of FLAG–NFAT5_132–581_ under both isotonic and hypotonic conditions (Fig. 4C). Among the other exportins, gene knockdown of XPOT and XPO4 mitigated hypotonicity-induced nuclear export of FLAG–NFAT5_132–581_ (Fig. 4C; Fig. S3B). Interestingly, we found that XPOT, which is the exportin for tRNAs (Kutay et al., 2000), undergoes distinct cytoplasmic and nuclear localization similar to that of NFAT5 in response to hypotonic and hypertonic treatments (Fig. 4D). These data suggest that XPOT is regulated by changes in extracellular tonicity and is a potential exportin for NFAT5 nuclear export. We further determined nucleocytoplasmic trafficking of endogenous NFAT5 upon gene knockdown of XPOT. Knockdown of XPOT significantly reduced the abundance of endogenous NFAT5 (Fig. S3C), presumably due to the reduced availability of tRNAs for protein translation. On the other hand, whereas knockdown of XPOT did not significantly alter the distribution of NFAT5 under isotonicity, it profoundly inhibited hypotonicity-induced NFAT5 nuclear export (Fig. 4E). However, co-immunoprecipitation of FLAG–NFAT5_132–581_ failed to pull down endogenous XPOT in the immunocomplex (Fig. S3D), presumably because of the transient nature of the exportin–cargo interaction.

### Identification and characterization of novel NFAT5-interacting proteins for nuclear export

**Fig. 5.**
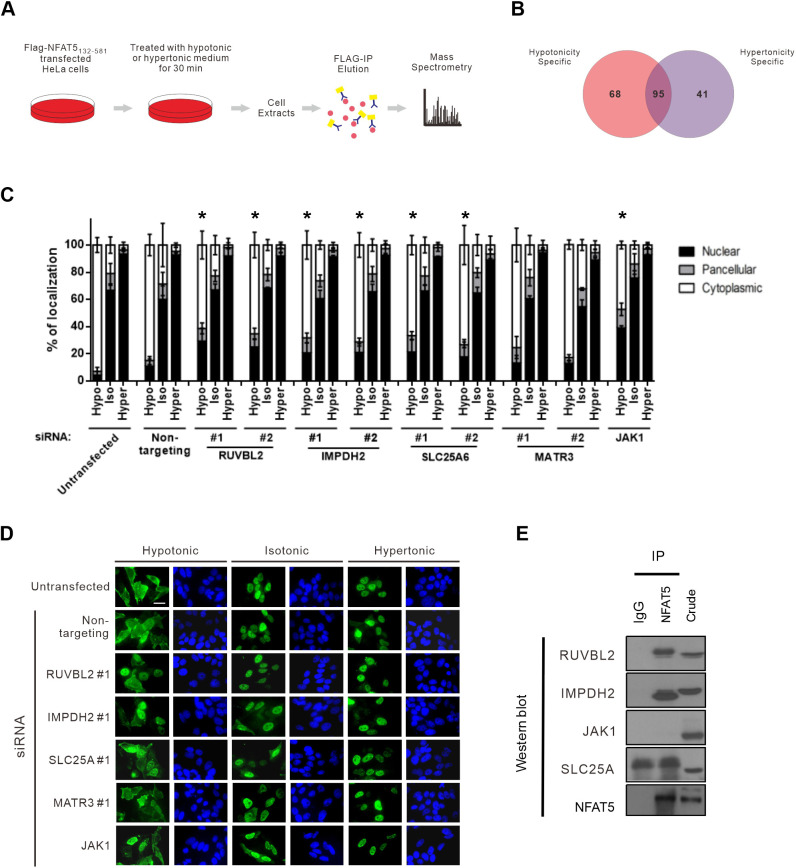
**Identification and characterization of novel NFAT5-interacting proteins for nucleocytoplasmic trafficking.** (A) Schematic of the experimental setup for proteomic identification of NFAT5-interacting proteins. HeLa cells expressing FLAG–NFAT5_132–581_ were switched to hypertonic or hypotonic medium for 30 min before harvesting. Cell extracts were subjected to immunoprecipitation using anti-FLAG antibodies (FLAG-IP). The immunocomplexes were digested with trypsin and Lys-C, and subjected to mass spectrometric analysis. The MS/MS spectra were subjected to SEQUEST for protein identification. (B) Venn diagram showing the number of proteins specifically associated with NFAT5 under hypotonic and hypertonic conditions, and the number of proteins associated with NFAT5 under both conditions. Data are from a single experiment. (C) Quantitative analysis of subcellular localization of immunofluorescence signal in HeLa cells transfected with FLAG–NFAT5_132–581_ and the indicated siRNA. Cells were switched to hypotonic (Hypo), isotonic (Iso) or hypertonic (Hyper) medium before fixation. For each condition, at least 100 cells were scored. Data are presented as mean±s.e.m. of three independent experiments. *P<0.05 indicates significant reduction in cytoplasmic fluorescence signal compared with that in the non-targeting siRNA-expressing cells under the same tonicity (one-way ANOVA with Bonferroni's multiple comparison test). (D) Representative fluorescence images of HeLa cells co-transfected with FLAG–NFAT5_132–581_ and the indicated siRNA. Cells were switched to hypertonic, isotonic or hypotonic medium for 90 min before fixation. Cells were stained with FLAG antibody and FITC-labeled secondary antibody (green, left-hand images), and were counterstained with DAPI (blue, right-hand images). Scale bar: 30 µm. Images are representative of three independent experiments. (E) Co-immunoprecipitation analysis of the putative NFAT5-interacting proteins. Immunoprecipitation (IP) was carried out using NFAT5 antibodies, and the immunocomplexes were subjected to western blot analysis using the indicated antibodies. IgG, IgG control; crude, crude extract. Blots shown are representative of three independent experiments.

The N-terminal region of NFAT5 is associated with many proteins (DuMond et al., 2016). To identify novel NFAT5-interacting proteins that might serve as potential NTRs or regulators for its nuclear export, we treated HeLa cells expressing FLAG–NFAT5_132–264_ with hypotonic or hypertonic medium, followed by affinity purification using FLAG antibodies and mass spectrometric analysis of the immunocomplexes (Fig. 5A; Tables S1 and S2). A total of 163 putative NFAT5 interactors were identified. Among them, 68 and 41 proteins were specifically associated with NFAT5 under hypotonic and hypertonic conditions, respectively, whereas 95 proteins were associated with NFAT5 under both conditions (common interactors) (Fig. 5B; Table S3).

A total of 95 putative NFAT5 interactors (hypotonicity-specific and common interactors) were selected for functional evaluation for their role in hypotonicity-induced NFAT5 nuclear export. Cytoskeletal proteins, histones, ribosomal proteins and mitochondrial proteins were excluded from the analysis because it is known that these proteins tend to associate with the immunocomplex in a non-specific manner. SMARTpool siRNAs were used to knock down the expression of each of these proteins, and the subcellular localization of FLAG–NFAT5_132–264_ was determined under different tonicities. We found that cells expressing siRNAs against RuvB-like AAA-type ATPase 2 (RUVBL2, also known as reptin), inosine-5'-monophosphate dehydrogenase 2 (IMPDH2), Janus kinase 1 (JAK1), adenine nucleotide translocator 3 (SLC25A6) or matrin 3 (MATR3) had profoundly reduced cytoplasmic localization of FLAG–NFAT5_132–264_ under hypotonic conditions (defined as less than 50% of the total FLAG signal being localized in the cytoplasm) (Fig. S4).

Secondary screening of each putative interactor using two independent siRNAs (Fig. S5) confirmed that hypotonicity-induced nuclear export of FLAG–NFAT5_132–264_ was significantly blocked by gene knockdown of RUVBL2, SLC25A6, IMPDH2 or JAK1 (for which one siRNA was used) (Fig. 5C,D). Furthermore, immunoprecipitation assays revealed that endogenous NFAT5 associates with RUVBL2 and IMPDH2, but not with SLC25A6 or JAK1 (Fig. 5E). The role of RUVBL2 was further characterized in this study because we observed a distinctive nuclear accumulation of the FLAG–NFAT5_132–264_ fusion protein when RUVBL2 was depleted (Fig. 5D).

### RUVBL2 is required for hypotonicity-induced NFAT5 nuclear export

**Fig. 6.**
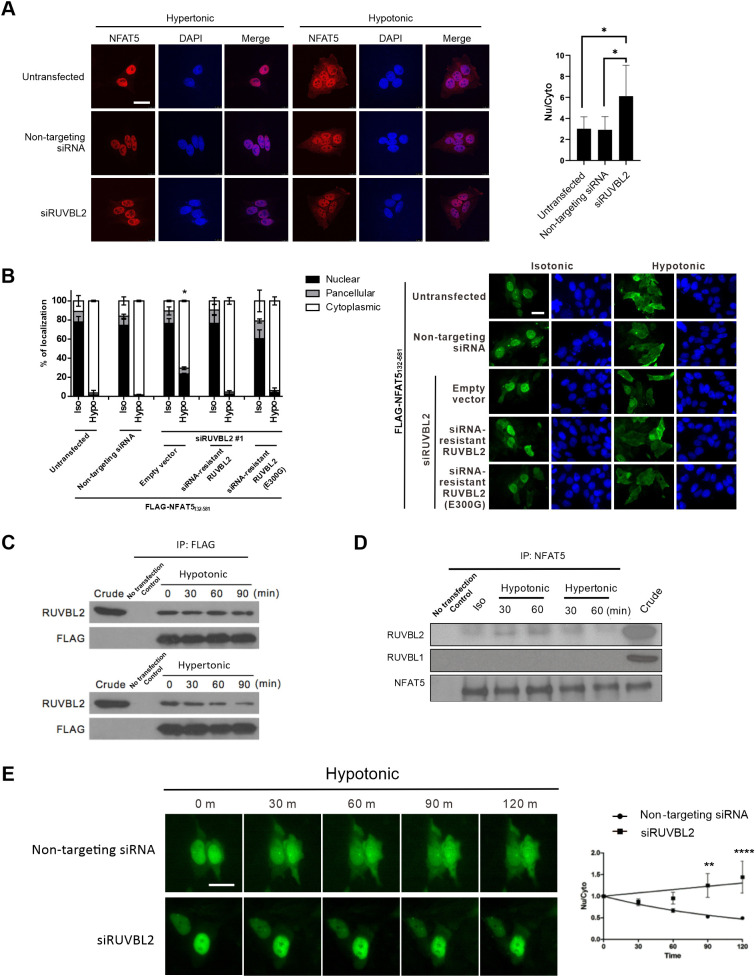
**Functional role of RUVBL2 in NFAT5 nuclear export.** (A) RUVBL2 knockdown inhibited nuclear export of NFAT5. Left: HeLa cells transfected with the indicated siRNA were treated with hypertonic or hypotonic medium for 90 min before fixation. Immunofluorescence was conducted using antibodies against NFAT5, followed by staining with FITC-labeled secondary antibodies. Cells were counterstained using DAPI. Scale bar: 30 μm. Right: quantitative analysis of endogenous NFAT5 subcellular localization under different siRNA treatments. The nuclear:cytoplasmic ratio of NFAT5 fluorescence signal (Nu/Cyto) in cells was calculated by measuring fluorescence intensity in the nucleus and cytoplasm using ImageJ. Data are presented as mean±s.e.m. of three independent experiments. *P<0.01 (one-way ANOVA with Bonferroni's multiple comparison test). (B) Rescue of RUVBL2 expression restored NFAT5 nuclear export. Left: quantitative analysis of subcellular localization of FLAG–NFAT5_132–581_ in cells co-transfected with RUVBL2 siRNA and with siRNA-resistant wild-type RUVBL2 or RUVBL2 E300G mutant. Cells were switched to isotonic (Iso) or hypotonic (Hypo) medium for 90 min before fixation. For each condition, at least 100 cells were scored. Data are presented as mean±s.e.m. of three independent experiments. *P<0.01 indicates significant induction in nuclear fluorescence compared with that of the non-targeting siRNA-expressing cells in the hypotonic condition (one-way ANOVA with Bonferroni's multiple comparison test). Right: representative fluorescence images are shown (FLAG–NFAT5_132–581_, green; DAPI, blue). Scale bar: 30 µm. (C) Interaction between NFAT5 and RUVBL2. Cell extracts prepared from HeLa cells expressing FLAG–NFAT5_132–581_ treated with the indicated tonicity were immunoprecipitated (IP) with FLAG antibodies. The immunocomplexes were subjected to western blotting using RUVBL2 and FLAG antibodies. Crude extract (5% total lysate) is shown as an input control. Blots are representative of three independent experiments. (D) NFAT5 was associated with RUVBL2 under isotonic and hypotonic conditions. HeLa cell extracts prepared from cells treated with the indicated tonicity were immunoprecipitated with NFAT5 antibodies. The immunocomplexes were subjected to western blot hybridization using the RUVBL2, RUVBL1 and NFAT5 antibodies. Crude extract (5% total lysate) is shown as an input control. Blots are representative of three independent experiments. (E) Live-cell monitoring of NFAT5–EGFP fusion protein trafficking in response to hypotonic treatment. HeLa cells were transfected with NFAT5_128–581_–EGFP and non-targeting siRNA, or with NFAT5_128–581_–EGFP and RUVBL2 siRNA (siRUVBL2). Cells were pre-treated with cycloheximide for 1 h and then induced with hypotonic medium, and EGFP fluorescence was captured at 0, 30, 60, 90 and 120 min. Left: representative images of GFP fluorescence. Scale bar: 30 µm. Right: quantification of Nu/Cyto fluorescence signal in cells transfected with non-targeting siRNA or RUVBL2 siRNA. Data are presented as mean±s.e.m. of three independent experiments (for each condition, *n*≥9 cells were analyzed). **P<0.01; ****P<0.0001 (two-way ANOVA with Bonferroni's multiple comparison test).

RUVBL2 and RUVBL1 (also known as pontin) are two closely related proteins belonging to the large AAA+ ATPase (ATPases associated with diverse cellular activities) superfamily that are characterized by an ATPase core domain with Walker A and Walker B motifs (Jha and Dutta, 2009). RUVBL2 and RUVBL1 can either form a complex with each other and function together (Izumi et al., 2010), or act independently of each other (Bauer et al., 2000; Rottbauer et al., 2002; Diop et al., 2008; Kim et al., 2005), to regulate a wide variety of functions, including chromatin remodeling, transcription regulation, DNA damage responses and assembly of ribonucleoprotein complexes (Izumi et al., 2010). We found that hypotonicity-induced nuclear export of endogenous NFAT5 (Fig. 6A) or FLAG–NFAT5_132–581_ (Fig. 6B) was significantly mitigated in cells transfected with RUVBL2 siRNA. Expression of siRNA-resistant RUVBL2 in cells transfected with RUVBL2 siRNA restored hypotonicity-induced nuclear export of FLAG–NFAT5_132–581_ (Fig. 6B), suggesting that RUVBL2 exerts a direct effect on NFAT5 nuclear export. RUVBL2 contains an ATPase domain that may be essential for its function (Grigoletto et al., 2011). Expression of an siRNA-resistant RUVBL2 ATPase mutant, RUVBL2 (E300G), restored NFAT5 nuclear export similar to the overexpression of siRNA-resistant RUVBL2 (Fig. 6B; Fig. S6A). Concordantly, we found that CB-6644, a small-molecule inhibitor of the ATPase activity of RUVBL1 and RUVBL2 (Assimon et al., 2019), failed to block hypotonicity-induced nuclear export of FLAG–NFAT5_132–264_ (Fig. S6B). These findings suggest that RUVBL2 regulates NFAT5 nuclear export in an ATPase-independent manner. However, siRNA-mediated knockdown of RUVBL1 (Fig. S6C) did not significantly alter NFAT5 nuclear export (Fig. S6D), suggesting that RUVBL2 does not cooperate with RUVBL1 to mediate the process. Furthermore, the results of co-immunoprecipitation assays suggested that FLAG–NFAT5_132–264_ and RUVBL2 consistently associate with each other, but also showed that the interaction between the two proteins is reduced under hypertonicity (Fig. 6C). Similarly, we observed reduced association between endogenous NFAT5 and RUVBL2 under hypertonic condition (Fig. 6D), which was not due to reduced expression of RUVBL2 under hypertonicity (Fig. S6E).

To further substantiate the function of RUVBL2 in NFAT5 nuclear export, we expressed an NFAT5–EGFP reporter protein (NFAT5_128–581_–EGFP) for real-time monitoring of its trafficking in cells expressing non-targeting siRNA or RUVBL2 siRNA, using time-lapse fluorescence microscopy as we have described previously (Xu et al., 2008). We found that hypotonicity induced time-dependent nuclear export of GFP signal in cells expressing non-targeting siRNA, but this export was remarkably inhibited in cells expressing RUVBL2 siRNA (Fig. 6E). Taken together, these data suggested that RUVBL2 is essential for nuclear export under hypotonicity.

### Nucleocytoplasmic trafficking of RUVBL2 in response to changes in extracellular tonicity

**Fig. 7.**
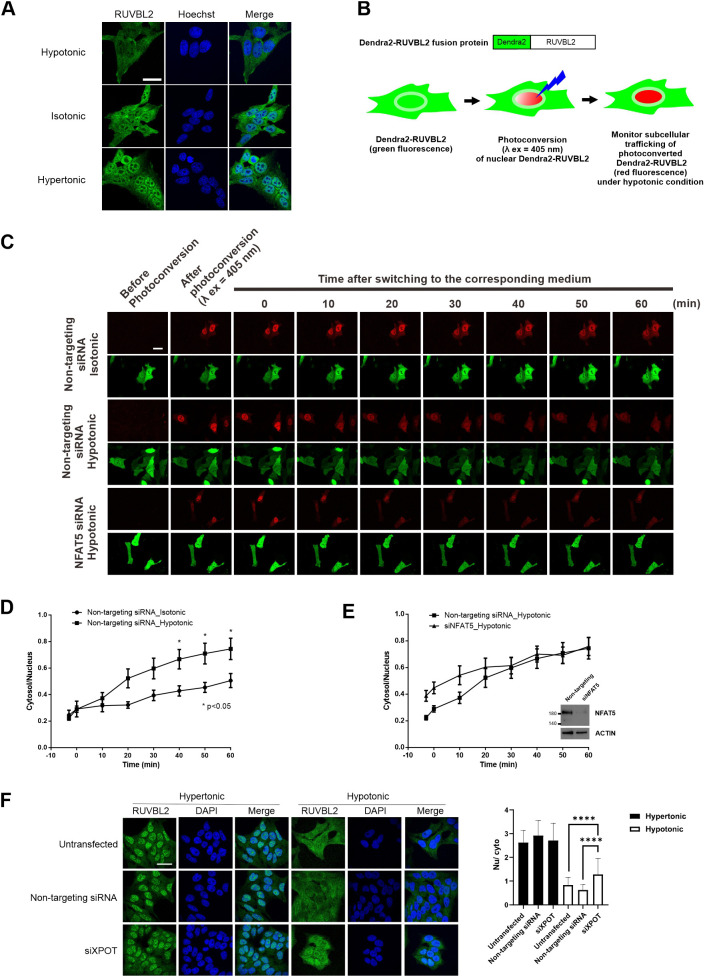
**Nucleocytoplasmic trafficking of RUVBL2 in response to changes in extracellular tonicity, and the role of NFAT5 and XPOT in the process.** (A) Representative immunofluorescence images of endogenous RUVBL2 localization in hypotonic, isotonic and hypertonic medium. HeLa cells were treated with the indicated tonicity for 90 min. RUVBL2 was visualized using an RUVBL2 antibody. Cells were counterstained with Hoechst. Scale bar: 30 µm. Images are representative of three independent experiments. (B) Schematics of the Dendra2–RUVBL2 experimental setup. HeLa cells expressing Dendra2–RUVBL2 and either non-targeting siRNA or NFAT5 siRNA were photoconverted using a 405 nm laser (λ ex, excitation wavelength). Dendra2–RUVBL2 in the nucleus was photoconverted from green to red. The Dendra2–RUVBL2 protein trafficking in live cells in response to hypotonic treatment was subsequently monitored. (C) Dendra2–RUVBL2 time-lapse experiment using HeLa cells transfected with non-targeting siRNA or NFAT5 siRNA. Top: representative red fluorescence images of photoconverted Dendra2–RUVBL2 fusion protein. Bottom: representative green fluorescence images of non-photoconverted Dendra2–RUVBL2 fusion protein in cells. After 405 nm photoconversion, cells were treated with hypotonic medium. Fluorescence images were captured every 10 min. Scale bar: 30 µm. (D,E) Quantification of the cytoplasmic:nuclear ratio of photoconverted Dendra2–RUVBL2 red fluorescence signals over time in (D) cells transfected with non-targeting siRNA and treated with isotonic or hypotonic medium, or (E) cells transfected with NFAT5 siRNA (siNFAT) or non-targeting siRNA and subjected to hypotonic stress. Data are presented as mean±s.e.m. of three independent experiments (for each condition, *n*≥10 cells were analyzed). *P<0.05 (two-way ANOVA with Bonferroni's multiple comparison test). Inset in E shows western blot analysis of NFAT5 expression in cells transfected with non-targeting and siNFAT5 siRNAs. Actin is shown as a loading control. (F) Left: representative immunofluorescence images of endogenous RUVBL2 localization in HeLa cells transfected with control or XPOT siRNA (siXPOT), under hypotonic and hypertonic conditions. Cells were counterstained using DAPI. Scale bar: 30 μm. Right: quantitative analysis of nucleocytoplasmic distribution of RUVBL2 under the indicated conditions. The nuclear:cytoplasmic ratio (Nu/cyto) of RUVBL2 fluorescence signal in cells was calculated by measuring fluorescence intensity in the nucleus and cytoplasm using ImageJ. At least 18 cells in each condition were scored. ****P<0.001 (one-way ANOVA with Tukey's multiple comparisons test).

RUVBL2 primarily resides in the nucleus (Bauer et al., 1998; Sigala et al., 2005). We first determined the subcellular localization of RUVBL2 under different extracellular tonicities. Endogenous RUVBL2 is localized pan-cellularly under isotonic conditions. It becomes predominantly localized to the cytoplasm and nucleus in response to hypotonicity and hypertonicity challenge, respectively (Fig. 7A), suggesting that RUVBL2 undergoes nucleocytoplasmic trafficking in response to changes in extracellular tonicity per se. To further understand the relationship between RUVBL2 and NFAT5, we created a Dendra2–RUVBL2 reporter construct (Fig. 7B). Dendra2 is a green-light-emitting fluorescent protein that can be converted to emit red light upon excitation at 405 nm (Gurskaya et al., 2006). Upon photoconversion, the Dendra2 reporter allows live-cell monitoring of the tagged protein without interference from newly synthesized or unconverted proteins (Fig. 7B) (Chudakov et al., 2007). Similar to the localization of endogenous RUVBL2, we observed that Dendra2–RUVBL2 is localized pan-cellularly (Fig. 7C, green fluorescence). Time-lapse confocal microscopy showed that, in cells transfected with non-targeting siRNA, photoconverted Dendra2–RUVBL2 remained resident in the nucleus after 60 min in isotonic conditions. Under hypertonic conditions, it underwent cytoplasmic translocation in a time-dependent manner, with a concomitant reduction in signal intensity (Fig. 7C,D). Remarkably, hypotoncity-induced nuclear export of photoconverted Dendra2–RUVBL2 was not affected by reduced availability of NFAT5 following transfection of NFAT5 siRNA (Fig. 7C,E). Taken together, our data suggest that RUVBL2 is required for hypotonicity-induced nuclear export of NFAT5, but not vice versa. Therefore, RUVBL2 is a chaperone for NFAT5 nuclear export. To further elucidate whether RUVBL2 nuclear export requires XPOT, we conducted siRNA-mediated knockdown of XPOT and monitored subcellular localization of RUVBL2 under both hypertonic and hypotonic stresses. Significant accumulation of RUVBL2 signal was found in the nucleus of cells expressing XPOT siRNA under hypotonic conditions (Fig. 7F). Taken together, these data support a role for XPOT as a putative nuclear export receptor for the NFAT5–RUVBL2 complex.

### Interactions between NFAT5 and RUVBL2

**Fig. 8.**
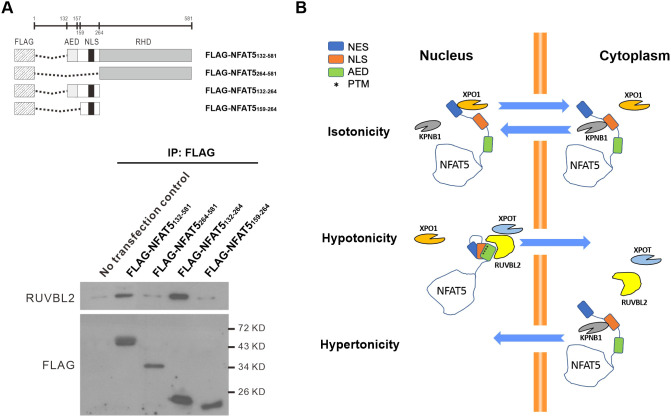
**Interaction between NFAT5 and RUVBL2.** (A) Top: schematics of FLAG–NFAT5 deletion constructs. Bottom: HeLa cells expressing the indicated NFAT5 deletion mutant constructs were immunoprecipitated (IP) using anti-FLAG affinity resin. The immunocomplexes were subjected to western blot analysis using RUVBL2 and FLAG antibodies. KD, kilodalton. Blots are representative of three independent experiments. (B) Schematic showing the proposed mechanisms of NFAT5 nuclear import and nuclear export under different extracellular tonicities.

To further understand whether and how NFAT5 and RUVBL2 interact, we expressed various FLAG–NFAT5 deletion mutants (Fig. 8A, top) for co-immunoprecipitation analysis. As expected, co-immunoprecipitation of FLAG–NFAT5_132–581_ resulted in the presence of endogenous RUVBL2 in the immunocomplex. However, RUVBL2 signal was abolished in the immunocomplex of FLAG–NFAT5_264–581_ (Fig. 8A, bottom). Similarly, RUVBL2 was associated with FLAG–NFAT5_132–264_, but it failed to form any complexes with FLAG–NFAT5_159–264_ (Fig. 8A, bottom). These data suggest that the AED is crucial for NFAT5 to interact with RUVBL2, and hence for NFAT5 nuclear export. To further determine whether RUVBL2 interacts with the AED directly, we expressed recombinant FLAG–NFAT5_131–249_ and His–RUVBL2 and tested their binding using an ITC assay. However, we failed to detect any interaction, even after repeated trials (Fig. S7), suggesting that the two proteins might not interact directly in this setting.

## DISCUSSION

NFAT5 was discovered two decades ago as the first transcription factor known to be involved in orchestrating cellular adaptive responses to hypertonic stress (Ko et al., 2000; Miyakawa et al., 1999), and it is the best characterized tonicity-regulated transcription factor to date. Hypertonic stress was once considered to be present only in the inner medulla of the kidneys (Burg et al., 2007), but emerging evidence has suggested that excessive Na^+^ accumulation in other tissue microenvironments, such as the skin (Jantsch et al., 2015) and the intervertebral disc (Sadowska et al., 2018), might activate NFAT5 and cause either protective (Machnik et al., 2009; Sadowska et al., 2018) or pathogenic (Matthias et al., 2019; Ma et al., 2020) outcomes. Although the contemporary view on how Na^+^ accumulation leads to NFAT5 activation in these tissues has been challenged recently (Rossitto et al., 2020), there is evidence suggesting that dysregulation of NFAT5 contributes to the pathogenesis of autoimmune diseases (Matthias et al., 2019; Aramburu and López-Rodríguez, 2019; Lee et al., 2019; Kleinewietfeld et al., 2013), inflammation (Aramburu and López-Rodríguez, 2019), hypertension and metabolic disorders (Jantsch et al., 2014).

While the physiological and pathological roles of NFAT5 have been firmly established, the molecular mechanisms underlying how NFAT5 activity is regulated remain largely elusive. Our study has generated multiple insightful findings into the molecular mechanism of tonicity-dependent regulation of NFAT5 activity. We provide direct evidence at the nanoscale of NFAT5 trafficking through the NPC, using an NFAT5–MiniSOG fusion protein assay coupled with electron microscopy. Although NFAT5 is proposed to function in a dimeric state (López-Rodríguez et al., 2001), our NFAT5–MiniSOG reporter construct, which lacks the putative dimerization domain, undergoes nucleocytoplasmic trafficking. Therefore, NFAT5 undergoes nucleocytoplasmic trafficking in monomeric form and becomes dimerized upon binding to the target sites. We have also made pivotal observations demonstrating that NFAT5 forms clusters in the nucleus under hypertonic conditions. This finding raises an interesting possibility that NFAT5-bound genomic regions might fold into chromatin domains to better coordinate gene expression, similar to how CTCF bridges genomic sites at a megabase scale (Merkenschlager and Nora, 2015).

Previous bioinformatics analysis has suggested that NFAT5 contains a putative protein domain exhibiting high sequence homology to a monopartite cNLS (Tong et al., 2006), and it is presumed that its nuclear import is mediated by a heterotrimeric Kapα–Kapβ–NFAT5 complex (Lott and Cingolani, 2011). Unexpectedly, we found that NFAT5 utilizes an unconventional mechanism for nuclear import in which KPNB1 alone serves as both the adaptor and the importin. Such KPNB1-driven nuclear import has been reported for a number of structurally diverse proteins, including SREBP-2 (Lee et al., 2003) and Snail-1 (Choi et al., 2014). KPNB1 shows conformational flexibility across its elongated superhelical structure, thus allowing it to effectively ‘wrap’ around structurally diverse sequences for tight and specific interaction (Wing et al., 2022).

To facilitate KPNB1-driven nuclear import, the respective cargo molecule must bind to KPNB1 directly. Indeed, most cargo molecules bind to their respective import adaptor very tightly (Wing et al., 2022) For NFAT5, our data shows that both NFAT5-NLS and the shorter NFAT5-cNLS bind to KPNB1 with moderate micromolar affinity *in vitro*. Additionally, although NFAT5-cNLS is sufficient for KPNB1 binding, nuclear import activity could only be elicited by the extended NFAT5-NLS *in vivo*. It is possible that the N- and C-terminal extensions of NFAT5-cNLS contain elements that enhance binding to KPNB1, especially upon hypertonicity. Future studies are needed to investigate this possibility.

Members of the NFAT family (NFAT1–5) are characterized by the presence of RHDs (Graef et al., 2001). It is hypothesized that distinctive genome recombination events during evolution have led to differential acquisition of different regulatory sequences, giving rise to tonicity-responsive NFAT5 and Ca^2+^-responsive NFAT1–4 (Graef et al., 2001), which are involved in diverse cellular functions ranging from T cell response to organogenesis (Zhu and McKeon, 2000). An NFAT5 homolog can be found in *Drosophila*, whereas NFAT1–4 are restricted to vertebrates, suggesting that NFAT5 has a more ancestral origin (Graef et al., 2001). Our findings therefore suggest that the classical nuclear import pathway has also evolved to differentiate the activation of NFAT1–4 from activation of NFAT5.

Furthermore, our data strongly suggest that XPOT, an exportin for nuclear export of tRNA (Arts et al., 1998), is a novel exportin for NFAT5. Therefore, NFAT5 is the first protein cargo reported for XPOT. XPOT per se undergoes tonicity-dependent nucleocytoplasmic trafficking, implicating its novel functional role in regulating cellular adaptation to changes in extracellular tonicity. Depletion of XPOT profoundly compromises hypotonicity-induced nuclear export of NFAT5, suggesting that it is the exportin for NFAT5. XPOT undergoes nuclear export when bound to tRNA and RanGTP (Kutay et al., 1998), suggesting that hypotonicity-induced nuclear export of NFAT5 might be coupled with tRNA export. Functional implications of tRNA export during hypotonicity, as well how XPOT recognizes NFAT5 for nuclear export, require further investigation. Similarly, whether XPOT mediates the nuclear export of other proteins essential for cellular adaptation to changes in tonicity also requires further study. Our findings further suggest that RUVBL2 is a potential candidate for mediating the interaction between NFAT5 and XPOT. RUVBL2 has previously been identified as an NFAT5 binding partner (DuMond et al., 2016), but its functional significance in NFAT5 regulation has not been elucidated. RUVBL2 and the closely related protein RUVBL1 can exist as monomers, homodimers, and homo- or hetero-hexamers (Niewiarowski et al., 2010). Numerous functions of these complexes have been proposed, including being a scaffold for protein–protein interactions (Dauden et al., 2021). Here, we have shown that RUVBL2 mediates NFAT5 nuclear export in an ATPase- and RUVBL1-independent manner, and that RUVBL2 is required for NFAT5 nuclear export, but not vice versa. Conversely, RUVBL2 per se undergoes tonicity-dependent nucleocytoplasmic trafficking in response to changes in tonicity, where its nuclear export is dependent on XPOT. Taken together, our data strongly suggest that RUVBL2 serves by connecting XPOT to NFAT5, which is consistent with its role as a protein scaffold (Dauden et al., 2021). Whether a monomeric or polymeric RUVBL2 is involved requires further investigation.

Nuclear availability of many transcription factors is regulated by differential exposure of NLS and NES sequences. Putting together findings from this and previous studies, we propose a mechanistic model for the regulation of NFAT5 nucleocytoplasmic trafficking (Fig. 8B). Under isotonicity, the NFAT5-NLS collaborates with the classical NES (amino acids 8–15), through binding to KPNB1 and XPO1, respectively, to maintain NFAT5 nucleocytoplasmic shuttling in a homeostatic state. The AED and NLS are located in close proximity within the intrinsically disordered region, which is well-known for its structural flexibility for differential interaction with proteins (Garza et al., 2009). A number of tonicity-dependent post-translational modifications (PTMs) critical for nucleocytoplasmic trafficking of NFAT5, such as phosphorylation of T135 and Y143 (Gallazzini et al., 2011, 2010), as well as of S155 and S188 (Tong et al., 2009), are located within this region. Therefore, specific PTMs induced by changes in tonicity could lead to conformational change in NFAT5, resulting in differential exposure of the NLS and AED that allows interaction with KPNB1 and RUVBL2–XPOT, respectively. Whether formation of the NFAT5–RUVBL2–XPOT complex is dependent on specific PTMs or tRNAs requires further investigation.

In conclusion, our study reports a novel mechanism involved in cellular adaptation to changes in extracellular tonicity in mammalian cells. Unexpectedly, we have identified several molecules that were previously not known to have a role in the regulation of tonicity-dependent nucleocytoplasmic trafficking of proteins. Our findings open new avenues for study of NFAT5 and could lead to the development of therapeutic strategies for diseases that are associated with NFAT5 dysregulation.

## MATERIALS AND METHODS

### Cell culture and transfections

HeLa cells (American Type Culture Collection, Manassas, VA) were maintained in minimal essential medium (Gibco) supplemented with 10% fetal bovine serum (Gibco), 1 mM sodium pyruvate, 2 mM L-glutamine (Gibco) and 1% penicillin–streptomycin (300 mosmol/kg H_2_O) (complete medium), and were confirmed to be free from mycoplasma by routine testing. For the identification of NFAT5 interacting partners, transfected cells were incubated for 24 h in a complete isotonic growth medium before switching to a hypertonic or hypotonic medium. Hypotonic (260 mosmol/kg H_2_O) and hypertonic (500 mosmol/kg H_2_O) media were prepared by supplementing 10% fetal bovine serum, 1 mM sodium pyruvate, 2 mM L-glutamine and NaCl to NaCl-deficient minimal essential medium (Invitrogen) to the desired osmolality. Medium osmolality was measured using a Vapro^®^ vapor pressure osmometer (Wescor).

For functional elucidation of NFAT5 interactors using siRNA knockdown, cells were first transfected with the corresponding SMARTpool siRNA (Dharmacon) using DharmaFECT (Dharmacon) for 48 h, followed by replating of the cells in three cell culture plates. After 1 day, cells were transfected with the FLAG–NFAT5_132–581_ cDNA overnight, followed by switching to isotonic medium, or switching to hypotonic or hypertonic medium for 30 min.

### Plasmids, antibodies, siRNAs and inhibitors

FLAG–NFAT5_132–581_ was constructed as described previously (Xu et al., 2008). Site-directed mutagenesis was conducted using a QuikChange site-directed mutagenesis kit (Agilent). FLAG-NFAT5–PEPCK constructs (FLAG–NFAT5_132–264_PEPCK and FLAG–NFAT5_198–217_PEPCK) were constructed by in-frame insertion of the corresponding NFAT5 and PEPCK-C sequences into pFLAG-CMV-2 (Sigma-Aldrich) mammalian expression vector. FLAG–SV40_NLS_–PEPCK was constructed by in-frame insertion of SV40_NLS_ (PKKKRKV) and PEPCK-C into the pFLAG-CMV-2 vector. FLAG–NFAT5 constructs (FLAG–NFAT5_264–581_, FLAG–NFAT5_132–264_, and FLAG–NFAT5_159–264_) for immunoprecipitation analysis were generated by cloning the corresponding fragment into the pFLAG-CMV-2 vector. NFAT5_174–250_–MiniSOG was constructed by in-frame insertion of NFAT5_171–250_ into pCDNA3.1-MiniSOG (Boassa et al., 2013). GST–NFAT5 constructs (GST–NFAT5_171–250_, GST–NFAT5_171–220_, GST–NFAT5_220–250,_ GST–NFAT5_190–250_, GST–NFAT5_190–217_, GST–NFAT5_190–217_) and GST–SV40_NLS_ were cloned by in-frame insertion of the corresponding NFAT5 fragments and SV40_NLS_ into the C terminus of GST of pGEX-4T-1 (GE Healthcare). Dendra2-Lifeact-7, FLAG-TIP49b (RUVBL2), pET-Ran(Q69L) and AcGFP1-N1 plasmids were obtained from Addgene. Dendra2–RUVBL2 was constructed by in-frame insertion of Dendra2 into the N-terminus of RUVBL2. NFAT5_128–581_–EGFP was constructed by in-frame insertion of EGFP to the C terminus of FLAG–NFAT5_128–581_. ATPase-deficient RUVBL2 was generated by introducing an E300G mutation into RUVBL2. siRNA-resistant wild-type RUVBL2 and siRNA-resistant RUVBL2 ATPase mutant (E300G) were constructed by site-directed mutagenesis of Flag-TIP49b (RUVBL2) using a QuikChange site-directed mutagenesis kit (Agilent). His–NFAT5_171–250_–AcGFP, His–SV40_NLS_–AcGFP, and His–KPNB1 were constructed by in-frame insertion of NFAT5_171–250_ and AcGFP, SV40_NLS_ and AcGFP, and KPNB1 into a modified pET 30a vector, respectively (Pan et al., 2017). Myc–Bimax1 and Myc–Bimax2 cDNA were inserted into the BamH1 and EcoR1 sites of pCMV vectors to generate the plasmids. His–KPNA5 protein was obtained from LifeSpan BioSciences, Inc. SMARTpool siRNAs for target validation of targets identified by mass spectrometric analysis, against individual Kaps (IPO4, IPO5, IPO7, IPO8, IPO9, IPO11, IPO13, KPNB1, TNPO1, TNPO3, SNUPN, KPNA1, KPNA2, KPNA3, KPNA4, KPNA5 and KPNA6), and siRNAs against RUVBL1, RUVBL2, JAK1, SLC25A6, MATR3 and IMPDH2, were from Dharmacon. RUVBL2 (ab36569), SLC25A6 (ab154007), and IMPDH2 (ab131158) antibodies were obtained from Abcam and were used at 1:1000. JAK1 antibody (06-272) was from Millipore and was used at 1:1000. FLAG antibody (F7425) was from Sigma and was used at 1:1000 (for western blotting) and 1:1000 (for immunofluorescence) dilution. NF-90 antibody (sc-136197) was from Santa Cruz Biotechnology and was used at 1:1000 dilution. α-Tubulin antibody (T5168) was from Sigma and was used at 1:1000 dilution. An NFAT5 antibody (AAS31388C) obtained from Antibody Verify was used at 1:1000 dilution (for western blotting), and an NFAT5 antibody (F-9, sc-398171) obtained from Santa Cruz Biotechnology was used at 1:100 (for western blotting) and 1:50 (for immunofluorescence) dilution. KPNB1 (MA3-070), exportin T (PA5-66095) and exportin 4 (PA5-65820) antibodies were from Thermo Fisher Scientific and were used at dilutions of 1:1000 (for western blotting) and 1:250 (immunofluorescence). β-actin antibody (A5441) was from Sigma and was used at 1:1000 (for western blotting). CB-6644 was obtained from MedChemExpress and was used at 1 μM concentration.

### Photooxidation and EM preparation of transfected HeLa cells

Transfected HeLa cells plated on glass-bottom culture dishes (MatTek) were fixed in 2.5% glutaraldehyde in 0.1 M cacodylate buffer, pH 7.4, for 1 h on ice, rinsed five times in cold cacodylate buffer and blocked for 30 min with 10 mM KCN, 20 mM aminotriazole, 50 mM glycine and 0.01% hydrogen peroxide in cacodylate buffer (blocking buffer). For MiniSOG photooxidation, the transfected cells were identified using a Leica SPE II inverted confocal microscope with an 63× objective. Diaminobenzidine (DAB; Sigma) was dissolved in 0.1 M HCl at a concentration of 5.4 mg/ml. Freshly prepared DAB working solution at 10-fold dilution in blocking buffer (pH 7.4) was added to the plate, and HeLa cells were illuminated with 450–490 nm light from a xenon lamp for 3–4 min until a light brown reaction product was observed in place of the green fluorescence of MiniSOG. Cells were then removed from the microscope, washed in cold cacodylate buffer and postfixed in 1% osmium tetroxide for 30 min on ice. After several washes in cold double-distilled water, cells were either en bloc stained with 2% aqueous uranyl acetate for 1 h to overnight at 4°C, or directly dehydrated in a cold graded ethanol series (20%, 50%, 70%, 90%, 100%) for 3 min each on ice, then rinsed once in room temperature with 100% ethanol and embedded in Durcupan ACM resin (Electron Microscopy Sciences). Sections were cut with a diamond knife at a thickness of 70–90 nm for thin sections and 250 nm for thick sections for electron tomography. Thin sections were examined using a JEOL 1200 EX operated at 80 kV.

### Electron tomography

After sections were glow discharged, colloidal gold particles (5 nm diameter) were deposited on each specimen side to serve as fiducial markers. For reconstruction, 4-tilt series of images were recorded at regular tilt (angular increments of 0.5° from −60° to +60° increments) and four different azimuthal sample orientation with a FEI Titan Halo electron microscope operated at 300 kV. Magnification was set to 11 k. Tilt series were recorded using a 4k×4k Ceta camera. Fine alignment of the projections and 3D iterative reconstructions were performed with a custom reconstruction package (Lawrence et al., 2006; Phan et al., 2017).

### Immunofluorescence and confocal microscopy

Cells were washed three times with phosphate-buffered saline (PBS) and fixed with 4% w/v paraformaldehyde for 15 min at 4°C, followed by permeabilization with absolute methanol for 2 min at room temperature. Primary antibodies against indicated proteins and corresponding secondary antibodies were used to label the cells. To visualize the nuclei, cells were stained with 4,6-diamidino-2-phenylindole (DAPI; Sigma) or Hoechst 33342 (Thermo Fisher Scientific). Images were viewed either with a Zeiss Axiovert 200 M fluorescence microscope (with 63× objective) or Leica TCS SP8 MP confocal microscope system (with 63× objective). Quantification of fluorescence signals in different subcellular compartments was conducted using our previously described method (Xu et al., 2008; Tong et al., 2006).

### Immunoaffinity purification, multidimensional protein identification technology, mass spectrometry and database searching

HeLa cells were transfected with FLAG–NFAT5_132–581_. At 24 h after transfection, cells were pre-treated with hypotonic medium (260 mosmol/kg H_2_O) for 60 min and switched to hypertonic medium (450 msomol/kg H_2_O) for 30 min, and then lysed with lysis buffer [50 mM Tris-HCl, 150 mM NaCl, 1 mM EDTA, pH 7.5, 1% Triton X-100, cOmplete protease inhibitor cocktail (Roche) and PhosSTOP phosphatase inhibitor cocktail (Roche)]. The recombinant protein was purified by affinity chromatography using anti-FLAG affinity resin (Sigma). The purified protein was precipitated using 20% trichloroacetic acid. The precipitate was washed twice with acetone, dried, and resuspended in 8 M urea and 100 mM Tris-HCl, pH 8.5. The solubilized protein was reduced by the addition of Tris(2-carboxyethyl)phosphine to 5 mM, followed by the carboxyamidomethylation of cysteines using 10 mM iodoacetamide. The concentration of urea was then diluted 2-fold (to 4 M) by the addition of an equal volume of 100 mM Tris-HCl, pH 8.5. Sequencing grade endoproteinase Lys-C (Roche Diagnostics) and modified trypsin (Promega) were added at ∼1:50 enzyme to substrate ratio (w/w) before incubation at 37°C for 4 and 12–16 h, respectively. The resulting peptides were extracted with 5% formic acid and redissolved into buffer A (5% acetonitrile with 0.1% formic acid). Data-dependent tandem mass spectrometry (MS/MS) analysis was performed using an LTQ-Orbitrap mass spectrometer (Thermo Fisher Scientific). Protein identification was done with Integrated Proteomics Pipeline (IP2; Integrated Proteomics Applications, Inc., San Diego, CA; http://www.integratedproteomics.com), using the ProLuCID algorithm to conduct the database search (Eng et al., 1994; Xu et al., 2015) and DTASelect2 to filter the results (Cociorva et al., 2007; Tabb et al., 2002). Tandem mass spectra were extracted into ms1 and ms2 files from raw files using RawExtract 1.9.9 (http://fields.scripps.edu/downloads.php) and then were searched against EBI-IPI human protein database (version 3_71, released on 03-24-2010; McDonald et al., 2004). To estimate peptide probabilities and false discovery rates accurately, we used a reverse decoy database containing the reversed sequences of all the proteins appended to the target database (DuMond et al., 2016). All searches were parallelized and performed on an Intel Xeon 80 processor cluster under the Linux operating system. The peptide mass search tolerance was set to 10 ppm for spectra acquired on the LTQ-Orbitrap instrument. The mass of the amino acid cysteine was statically modified by +57.02146 Da to take into account the carboxyamidomethylation of the samples, and two peptides per protein and at least one trypitic terminus were required for each peptide identification. The ProLuCID search results were assembled and filtered using the DTASelect program (version 2.0) with false discovery rate of 0.05; under such filtering conditions, the estimated false discovery rate was below ∼1% at the protein level in all analyses.

### Live-cell microscopy and time-lapse imaging

Live-cell microscopy and time-lapse imaging were conducted as described previously (Xu et al., 2008). HeLa cells transiently expressing NFAT5_128–581_–EGFP fusion proteins were viewed with an inverted Zeiss fluorescence microscope using an 63× objective. A heated chamber perfused with CO_2_ was used to incubate the cells at 37°C. After pre-treatment with cycloheximde for 1 h (5 μg/ml; Tong et al., 2006), the original growth medium was removed and replaced with a pre-warmed hypotonic growth medium. Images were taken at 30 min intervals and analyzed using NIH ImageJ software (http://imagej.nih.gov/ij/). Quantification of signal intensity was conducted according to our previously described method (Ramírez et al., 2009). A circular region of interest (ROI; 25 pixels in diameter) was selected within the nucleus and cytoplasm of each cell for the quantification of signal intensity. The average background signal intensity was then subtracted from the average signal intensity.

### Photoconversion of Dendra2 and analysis of nuclear export of Dendra2–RUVBL2

Photoconversion of Dendra2–RUVBL2 from green to red fluorescence was achieved by exposing the cells to blue light according to a previously described method (Gurskaya et al., 2006). The cell nucleus was defined and exposed to 405 nm laser light for 4–5 bursts of 200 ms at ∼5% 405 nm laser power (TCS SP8 MP, Leica). After photoconversion of Dendra2–RUVBL2, the original growth medium was removed and replaced with a pre-warmed hypotonic growth medium. Images were taken at 10 min intervals. Post-acquisition image analyses were performed using NIH ImageJ software (http://imagej.nih.gov/ij/). A circular ROI (12 pixels in diameter) was selected within the nucleus and cytoplasm of each cell for the quantification of signal intensity. The average background signal intensity was then subtracted from the average signal intensity.

### Protein extraction, western blotting and immunoprecipitation

Cell protein lysates were obtained from cell cultures using lysis buffer (50 mM Tris-HCl pH7.8, 100 mM NaCl, 1 mM EDTA, 1% Triton X-100, 1 mM phenylmethylsolfonyl fluoride and 10 mM dithiothreitol). Cytoplasmic and nuclear extracts were prepared using Nuclear and Cytoplasmic Extraction reagents (Thermo Fisher Scientific). SDS–PAGE electrophoresis, western blotting and immunoprecipitation were conducted as described previously (Chen et al., 2013).

### Protein expression and purification

Full-length KPNB1 and various NFAT5 fragments were cloned into expression vectors with GST, 6×His or Trx–6×His–3C fusion tags and transformed into *E. coli BL21 (DE3)* bacteria (Invitrogen). Bacteria were harvested, lysed in lysis buffer (PBS with 5% glycerol, 100 mM MgCl_2_, 1 mM PMSF, 1 mM DTT, 1 mg/ml lysozyme, 0.2 units/ml DNase I, and Roche cOmplete protease inhibitors) and centrifuged (14,500 ***g***, 30 min) to remove debris. GST fusion proteins were purified from lysates with glutathione Sepharose 4 Beads (GE Healthcare). His-tag fusion proteins were purified from lysates using a HisTrap HP column (GE Healthcare). The size and purity of the protein preparations were verified by SDS-PAGE and Coomassie Blue staining. The Trx–6×His–3C tag was removed by protease 3C cleavage and the untagged protein was further purified by size-exclusion chromatography (Superdex 75, GE Healthcare).

### Isothermal titration calorimetry

Isothermal titration calorimetry (ITC) was performed using the MicroCal™ PEAQ-ITC (Malvern Panalytical Ltd). NFAT5_151–216_, NFAT5_189-–216_ and full-length KPNB1 were dialyzed into 50 mM Tris-HCl, pH 8.0, and 150 mM NaCl. NFAT5_171–253_ and full-length KPNB1 were dialyzed into 10 mM Na_2_HPO_4_·7H2O, 1.8 mM KH_2_PO_4_, 2.7 mM KCl, pH 7.4, and 150 mM NaCl. 150 μl of NFAT5_151–216_ or NFAT5_189–216_ protein at 1.5–1.75 mM was titrated into a sample cell loaded with 400 μl of full-length KPNB1 at 50 μM. 150 μl of NFAT5_171–253_ protein at 600 μM was titrated into a sample cell containing 400 μl of 20 μM KPNB1. Typically, titrations consisted of 19 injections of 2 μl, with 150 s equilibration between injections. The data were analyzed using MicroCal PEAQ-ITC Analysis Software.

### *In vitro* pulldown assay

GST fusion proteins, His–KPNB1 and Ni-NTA agarose (Invitrogen) were mixed and incubated at 4°C with shaking overnight. The mixtures were centrifuged at 2348 ***g***, 4°C for 2 min. The flow through was discarded. Agarose was washed three times with buffer (20 mM sodium phosphate, 100 mM sodium chloride, 40 mM imidazole, 10% glycerol). Proteins were eluted from the agarose by boiling in SDS loading buffer (100 mM Tris-HCl pH 6.8, 4% SDS, 20% glycerol, 2% β-mercaptoethanol, 25 mM EDTA, 0.04% Bromophenol Blue) for 10 min. After spinning down the debris, the supernatant was analyzed by SDS–PAGE and visualized using Coomassie Blue staining.

### Reconstitution of nuclear import in permeabilized cells

The assay was conducted as described previously (Cassany and Gerace, 2009). In brief, HeLa cells were grown in 10-well slides (Millipore). Cells were washed three times with cold transport buffer (20 mM HEPES, pH7.3, 110 mM potassium acetate, 2 mM magnesium acetate, 1 mM EGTA, 2 mM DTT, 1 mM PMSF, cOmplete protease inhibitor) on ice, followed by permeabilization with 50 µg/ml digitonin at room temperature for 5 min. Permeabilized cells were washed three times with cold transport buffer. The reaction was carried out in the presence of ATP-regenerating system [1 mM ATP, 1 mg/ml creatine phosphate (CP), and 15 U/ml creatine phosphate kinase (CPK); Calbiochem], 0.1 mM GTP, recombinant proteins (His–KPNB1, His–KPNA5, NTF2, Ran, His–NFAT5–AcGFP) and various supplements whenever necessary (0.8 mg/ml WGA, Sigma; 2 mg/ml cytosol preparation; 0.2 mM GTPγS, Calbiochem). After incubation, the cells were washed three times with cold transport buffer on ice and fixed with 4% paraformaldehyde on ice for 10 min, followed by permeabilization with 100% methanol for 5 min at room temperature. Nuclei were stained using DAPI for 5 min. After washing, cells were mounted on a glass slide using FluorSave™ Reagent (Millipore). The GFP and DAPI signals were observed using confocal microscope (Leica TCS SP8) with 63× objective.

### Statistical analysis

Statistical analysis was conducted using GraphPad Prism software. All data are expressed as mean±s.e.m. of triplicate experiments. Statistical significance was determined by unpaired *t*-test (two-tailed) for comparisons of two groups or by one-way ANOVA for comparisons of three or more groups, followed by Bonferroni's multiple comparison test as post test. Significance is shown in the figures; *P*<0.05 was considered statistically significant.

## Supplementary Material

Supplementary information

Reviewer comments
