# Supplementary material for: Unconventional tonicity-regulated nuclear trafficking of NFAT5 mediated by KPNB1, XPOT and RUVBL2
Source: J Cell Sci. 2022 Jul 12;135(13):jcs259280. doi: 10.1242/jcs.259280 (PMC9377714; doi:10.1242/jcs.259280)
Supplement: Supplementary information [file joces-135-259280-s1.pdf]

## Fig. S1

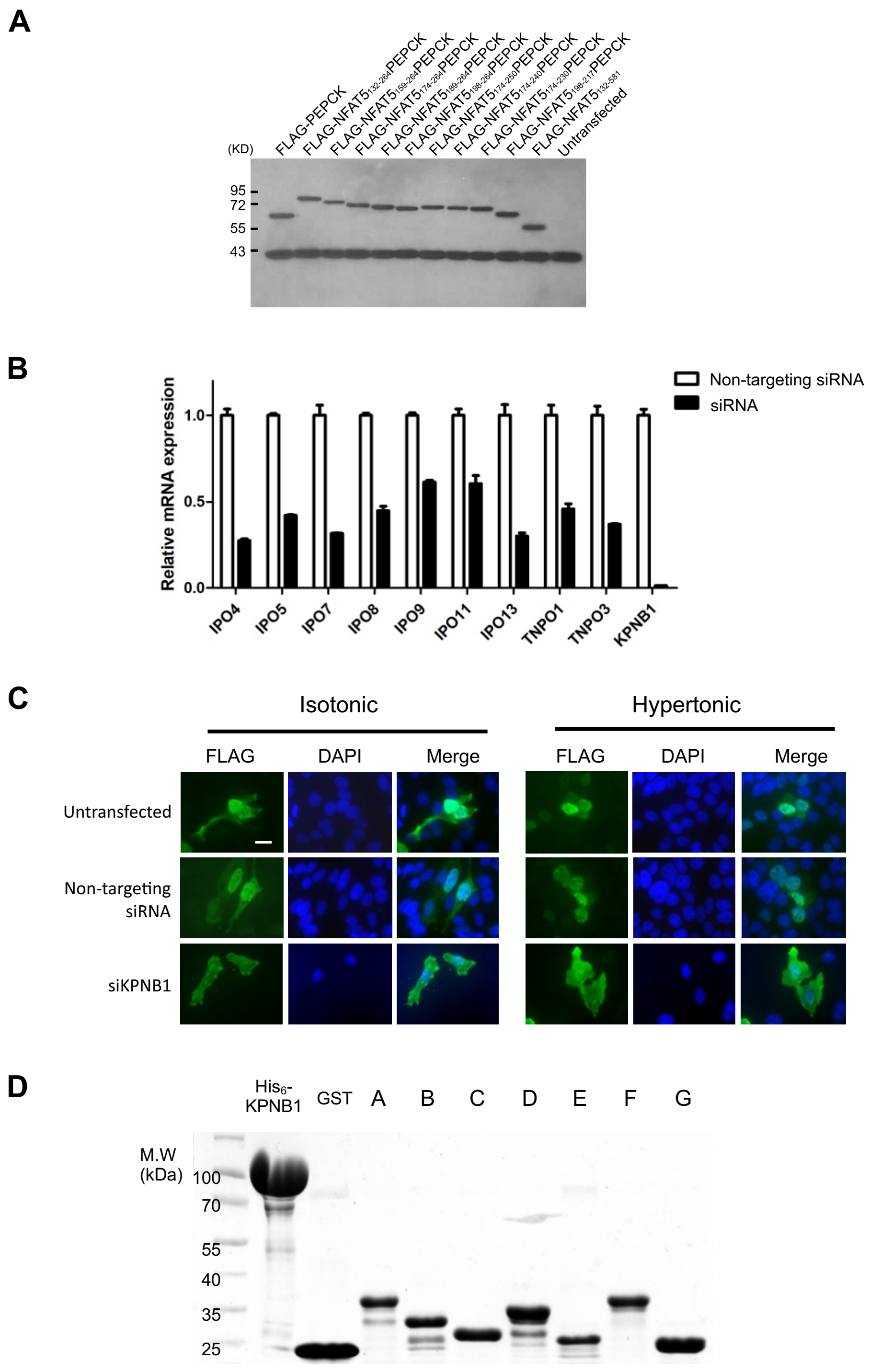


Journal of Cell Science • Supplementary information

**Figure S1. A)** Western blot analysis of FLAG-NFAT5$_{132\text{-}581}$ and FLAG-NFAT5-PEPCK mutants using FLAG antibodies. **B)** Real-time quantitative PCR analysis of the expression of the indicated genes in non-targeting siRNA- and gene-specific siRNA-transfected cells. **C)** Representative immunofluorescence images of FLAG-NFAT5$_{132\text{-}581}$ in the cells expressing the KPNB1 siRNAs. Immunofluorescence was carried out using FLAG antibodies followed by FITC-labelled secondary antibodies, and counterstained with DAPI. Scale bar is 30 µm. **D)** Recombinant GST-NFAT5 proteins from Fig. 1F after purification using glutathione sepharose column. Purified proteins were subjected to SDS-PAGE analysis followed by Coomassie blue staining. All proteins were expressed at the expected molecular weight.

**Fig. S2**

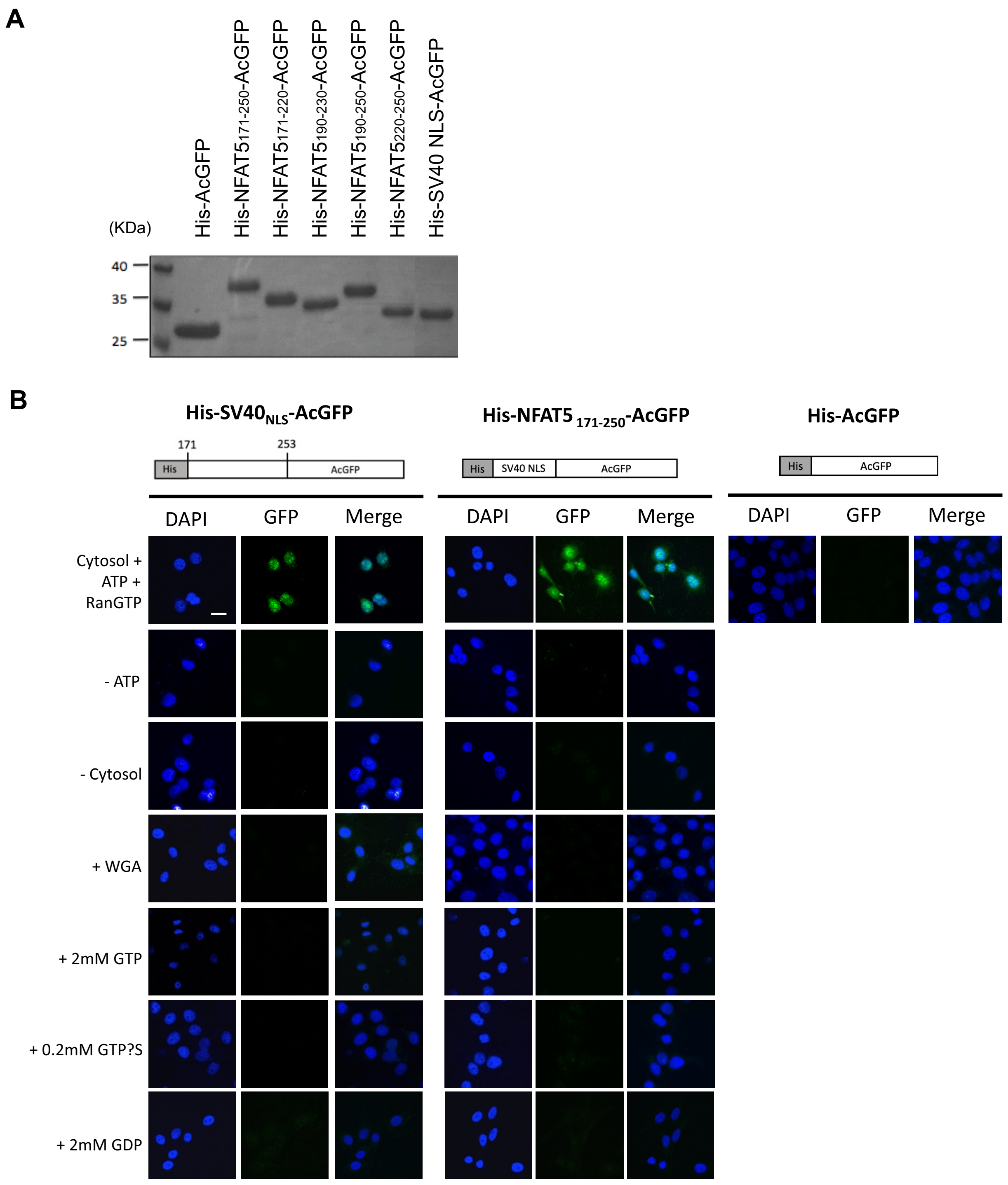
A
(KDa)
His-AcGFP
His-NFAT5171-250-AcGFP
His-NFAT5171-220-AcGFP
His-NFAT5190-230-AcGFP
His-NFAT5190-250-AcGFP
His-NFAT5220-250-AcGFP
His-SV40 NLS-AcGFP
40
35
25
B
His-SV40NLS-AcGFP
His-NFAT5 171-250-AcGFP
His-AcGFP
171
253
His
AcGFP
SV40 NLS
DAPI
GFP
Merge
Cytosol + ATP + RanGTP
- ATP
- Cytosol
+ WGA
+ 2mM GTP
+ 0.2mM GTP?S
+ 2mM GDP

**Fig. S2. A)** SDS-PAGE analysis of purified bacterially expressed recombinant proteins. Recombinant His-NFAT5-AcGFP proteins were purified using HisTrap column. All purified proteins were subjected to SDS-PAGE analysis and visualized by Coomassie blue staining. **B)** *In vitro* nuclear import assay. Nuclear import of His-SV40$_{NLS}$-AcGFP, His-NFAT5$_{171-250}$-AcGFP, and His-AcGFP were determined using digitonin permeabilized HeLa cells. Transport assay was carried out for 30 min at 37$^{o}$C, cells were fixed with paraformaldehyde, stained with DAPI and images were taken using confocal microscopy. Scale bar is 30 µm.

Journal of Cell Science • Supplementary information

## Fig. S3

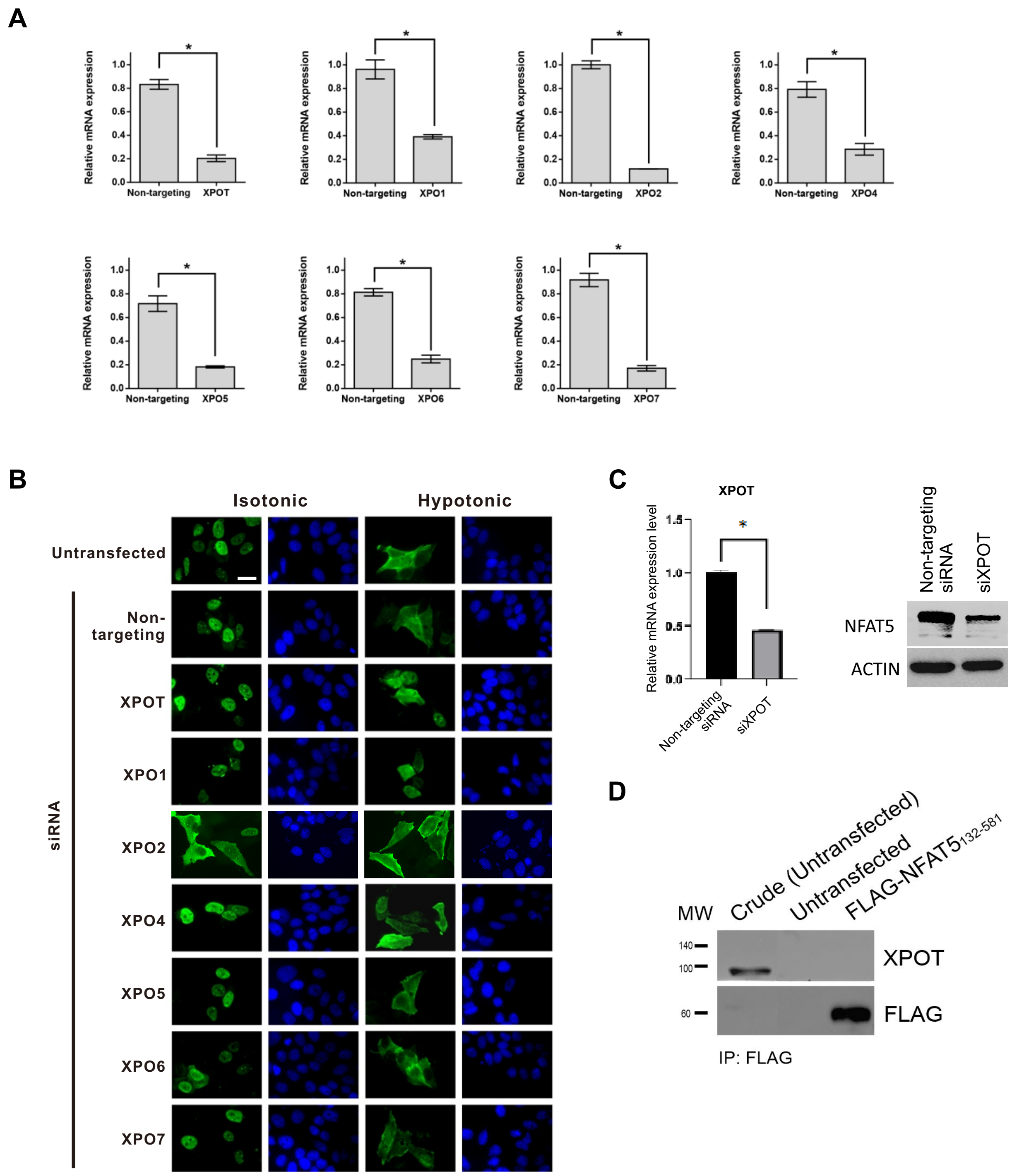


Journal of Cell Science • Supplementary information

**Figure S3.** siRNA analysis of exportins. **A)** Quantitative real-time PCR analysis of the exportins siRNA knockdown efficiency. Data are presented in mean ± SEM; *, $p < 0.05$, by unpaired t-test. **B)** Representative immunofluorescence images of HeLa cells transfected with FLAG-NFAT5$_{132\text{-}581}$ and the indicated siRNA. Cells were switched to isotonic or hypotonic medium for 90 mins before fixation. Cells were stained with FLAG antibody and FITC-labeled secondary antibody, counterstained with DAPI. Scale bar represents 30 µm. **C)** Left, quantitative real-time PCR analysis of the XPOT siRNA knockdown efficiency. Data are presented in mean ± SEM. *, p< 0.05. Right, Western blotting analysis of NFAT5 expression in HeLa cells transfected with non-targeting and XPOT siRNA respectively. **D)** Co-immunoprecipitation analysis of FLAG-NFAT5132-581 and XPOT. HeLa cells expressing FLAG-NFAT5132-581 prepared were treated with hypotonicity for 30 min, followed by immunoprecipitation with FLAG antibodies. The immunocomplexes were subjected to Western blot analysis using the XPOT antibodies.

Journal of Cell Science • Supplementary information

# Fig. S4

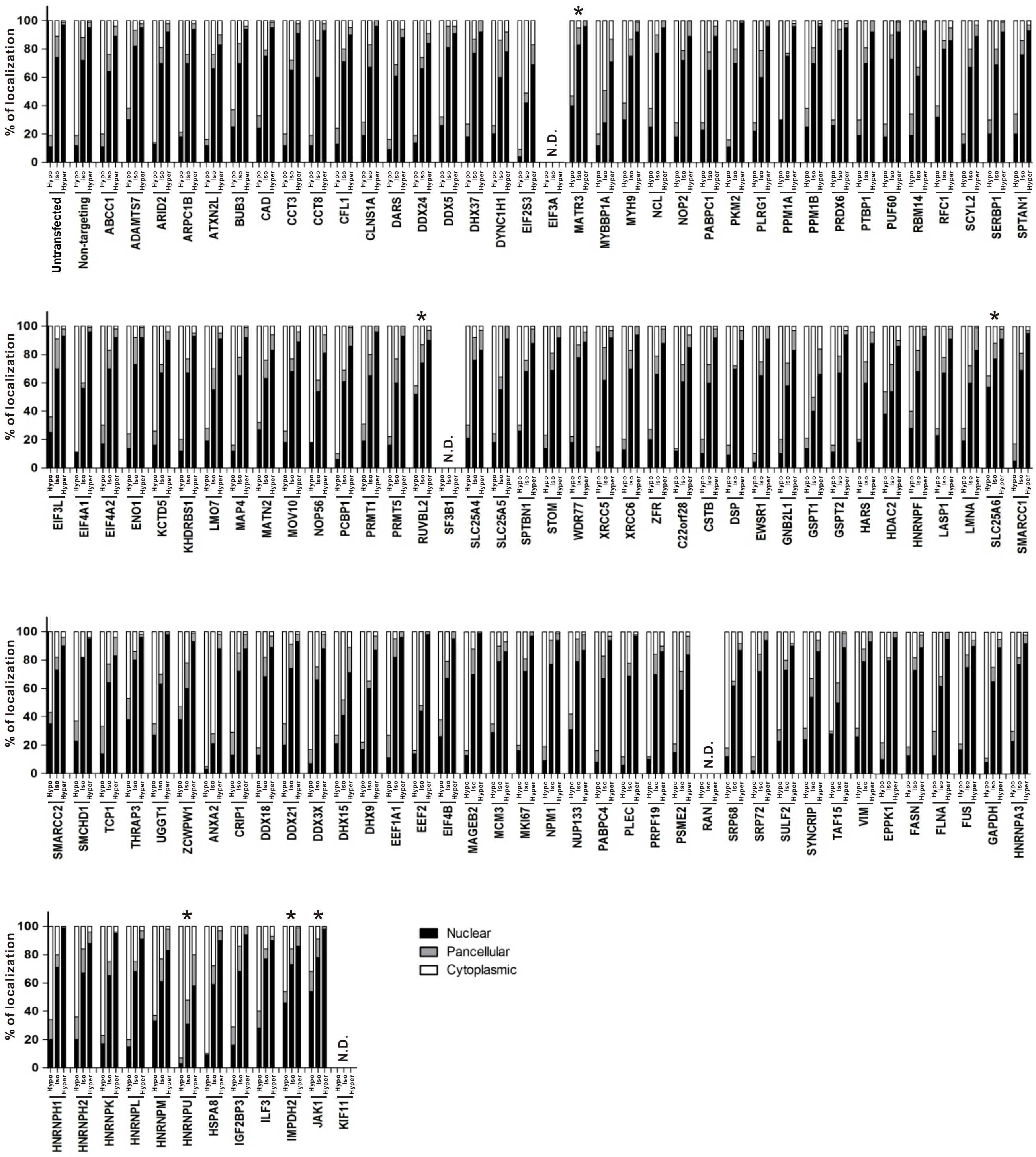


Journal of Cell Science • Supplementary information

**Fig. S4.** Quantification of the subcellular localization of FLAG- $NFAT5_{132-581}$ in HeLa cells in response to the expression of the indicated SMARTpool siRNAs library. Cells were treated with hypotonic (Hypo), isotonic (Iso) or hypertonic (Hyper) medium for 90 mins. Recombinant protein was visualized with FLAG antibodies and FITC-labeled secondary antibodies, counterstained with DAPI, and analyzed by fluorescence microscopy. For each condition, 100 cells were counted. *, $p<0.01$, by one-way ANOVA, between cytoplasmic FLAG signal in cells transfected with the indicated siRNA and the non-targeting SMARTpool siRNA under hypotonic condition.

Journal of Cell Science • Supplementary information

**Fig. S5**

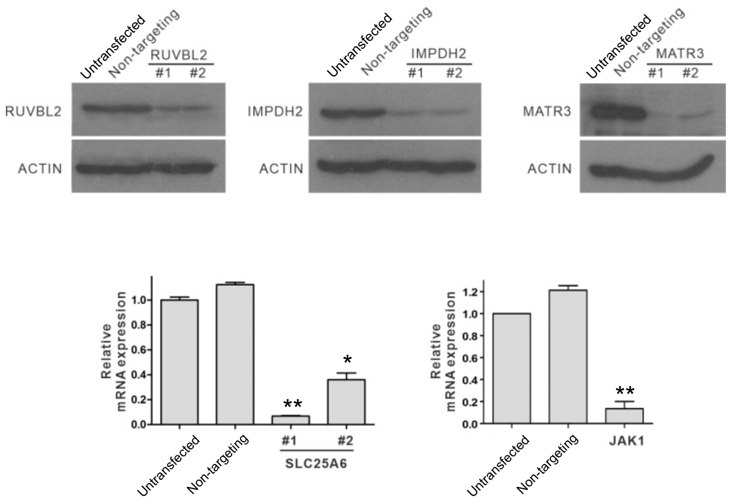


**Fig. S5.** Western blot analysis and quantitative real-time PCR analysis of the siRNA knockdown efficiency of the indicated putative target genes. Data of qPCR are presented in mean ± SEM; *, $p < 0.05$, **, $p < 0.01$, by one-way ANOVA with Bonferroni's multiple comparison test as post-test.

Journal of Cell Science • Supplementary information

**Fig. S6**

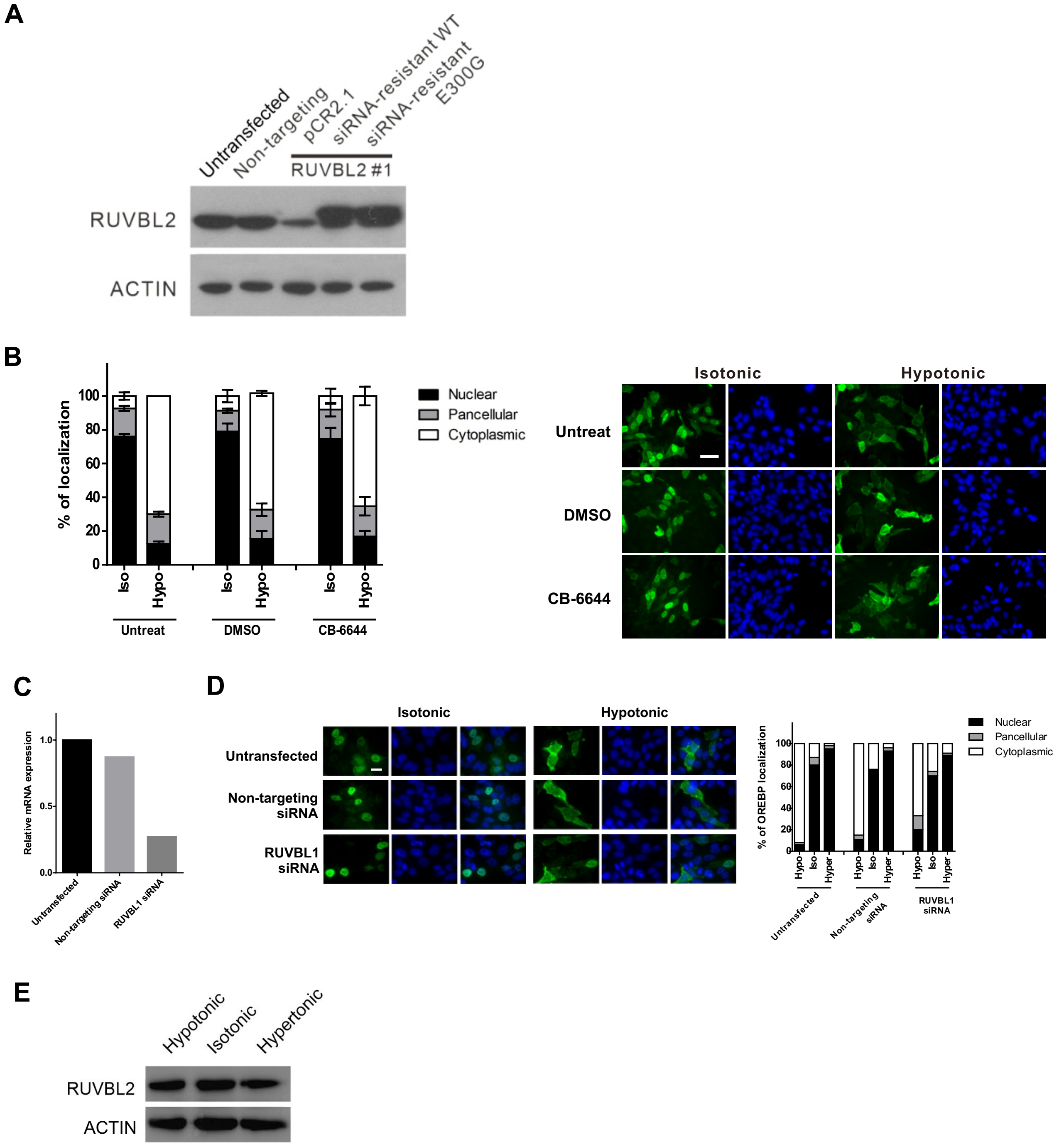


Journal of Cell Science • Supplementary information

**Fig. S6. A)** Western blot analysis showing the expression of endogenous RUVBL2, and recombinant siRNA-resistant wild-type (WT) RUVBL2 or siRNA-resistant RUVBL2 ATPase mutant (E300G). **B)** Left, quantitative analysis of the subcellular localization of FLAG-NFAT5$_{132\text{-}581}$ in cells treated with CB-6644. Cells were pre-treated with CB-6644 for 2h, followed by switching to isotonic or hypotonic medium in the presence or absence of CB-6644. Iso, isotonic condition; Hypo, hypotonic condition. Right, representative immunofluorescence cell images were shown, scale bar is 60 µm. **C)** Relative expression of RUVBL1 in HeLa cells transfected with non-targeting siRNA and RUVBL1 siRNA. **D)** Effect of RUVBL1 knockdown on NFAT5 nucleocytoplasmic trafficking. Left, representative fluorescence images of FLAG-NFAT5$_{132\text{-}581}$ in HeLa cells transfected with non-targeting siRNA or RUVBL1 siRNA. Cells were counterstained with DAPI. Scale bar is 30 µm. Right, quantitative analysis of siRNA knockdown of RUVBL1 in the subcellular localization of FLAG-NFAT5$_{132\text{-}581}$. Cells were treated with hypotonic, isotonic or hypertonic medium respectively. For each condition, at least 100 cells were counted. Hypo, hypotonic condition; Iso, isotonic condition; Hyper, hypertonic condition. Bottom right, the quantitative real-time PCR analysis of the siRNA knockdown efficiency of the indicated putative target genes. **E)** Western blot analysis indicates the endogenous expression level of RUVBL2 under different tonicities. Cells were treated with hypotonic, isotonic or hypertonic medium for 90 mins.

Journal of Cell Science • Supplementary information

**Fig. S7**

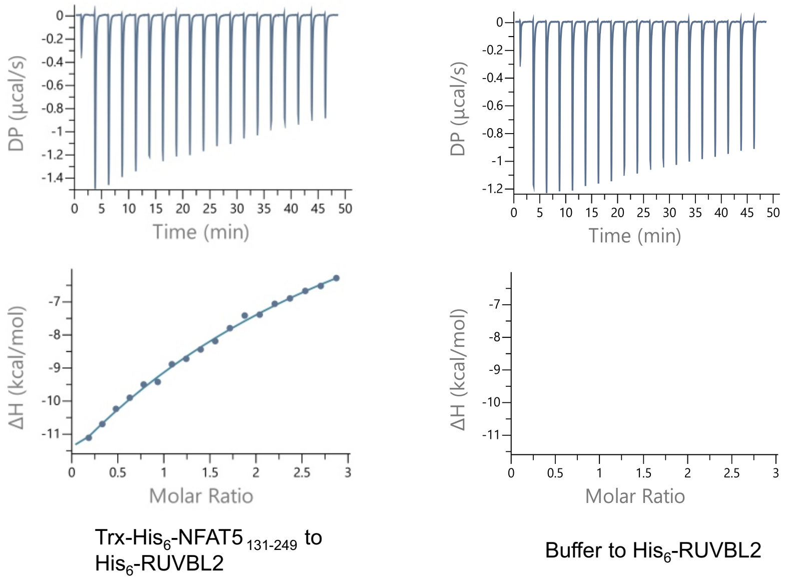


**Fig. S7.** Isothermal Titration Calorimetry (ITC) profiles to measure the binding affinity of NFAT5 to RUVBL2.

Journal of Cell Science • Supplementary information

**Table S1.** NFAT5 interacting proteins identified by mass spectrometric analysis at hypotonic condition.

Click here to download Table S1

**Table S2.** NFAT5 interacting proteins identified by mass spectrometric analysis at hypertonic condition.

Click here to download Table S2

**Table S3.** Summary of NFAT5 interacting proteins identified by mass spectrometric analysis. Protein candidates in the shaded boxes are either mitochondria, ribosomal, cytoskeletal proteins or histones, and were not included in the siRNA screen.

Click here to download Table S3
