# Supplementary material for: Unconventional tonicity-regulated nuclear trafficking of NFAT5 mediated by KPNB1, XPOT and RUVBL2
Source: J Cell Sci. 2022 Jul 12;135(13):jcs259280. doi: 10.1242/jcs.259280 (PMC9377714; doi:10.1242/jcs.259280)
Supplement: Reviewer comments [file joces-135-259280_review_history.pdf]

Unconventional tonicity-regulated nuclear trafficking of NFAT5 mediated by KPNB1, XPOT and RUVBL2

Chris Y. Cheung, Ting-Ting Huang, Ning Chow, Shuqi Zhang, Yanxiang Zhao, Mary P. Chau, Wing Cheung Chan, Catherine C. L. Wong, Daniela Boassa, Sebastien Phan, Mark H. Ellisman, John Yates, III, SongXiao Xu, Zicheng Yu, Yajing Zhang, Rui Zhang, Ling Ling Ng and Ben C. B. Ko
DOI: 10.1242/jcs.259280

Editor: Maria Carmo-Fonseca

Review timeline

Original submission:	18 August 2021
Editorial decision:	1 November 2021
First revision received:	2 May 2022
Accepted:	20 May 2022

Original submission

First decision letter

MS ID#: JOCES/2021/259280

MS TITLE: Unconventional tonicity-regulated nuclear trafficking of NFAT5 mediated by KPNB1, XPOT and RUVBL2

AUTHORS: Chris Y. Cheung, Ting-Ting Huang, Ning Chow, Shuqi Zhang, Yanxiang Zhao, Catherine CL Wong, Daniela Boassa, Sebastien Phan, Mark H Ellisman, John Yates, SongXiao Xu, Zicheng Yu, Yajing Zhang, Rui Zhang, Ling Ling Ng, and Ben Chi Bun Ko
ARTICLE TYPE: Research Article

We have now reached a decision on the above manuscript.

To see the reviewers' reports and a copy of this decision letter, please go to: https://submit-jcs.biologists.org and click on the 'Manuscripts with Decisions' queue in the Author Area. (Corresponding author only has access to reviews.)

As you will see, the reviewers raise a number of substantial criticisms that prevent me from accepting the paper at this stage. They suggest, however, that a revised version might prove acceptable, if you can address their concerns. Please address the issues raised by the the reviewers as thoroughly as possible. If you think that you can deal satisfactorily with the criticisms on revision, I would be pleased to see a revised manuscript. I would then return it to the reviewers.

We are aware that you may be experiencing disruption to the normal running of your lab that makes experimental revisions challenging. If it would be helpful, we encourage you to contact us to discuss your revision in greater detail. Please send us a point-by-point response indicating where you are able to address concerns raised (either experimentally or by changes to the text) and where you will not be able to do so within the normal timeframe of a revision. We will then provide further guidance. Please also note that we are happy to extend revision timeframes as necessary.

© 2022. Published by The Company of Biologists under the terms of the Creative Commons Attribution License (https://creativecommons.org/licenses/by/4.0/).

Please ensure that you clearly highlight all changes made in the revised manuscript. Please avoid using 'Tracked changes' in Word files as these are lost in PDF conversion.

I should be grateful if you would also provide a point-by-point response detailing how you have dealt with the points raised by the reviewers in the 'Response to Reviewers' box. Please attend to all of the reviewers' comments. If you do not agree with any of their criticisms or suggestions please explain clearly why this is so.

Reviewer 1

Advance summary and potential significance to field

The manuscript by Cheung et al. explored the mechanism of nucleocytoplasmic transport of NFAT5, which is regulated by extracellular tonicity. Authors demonstrated that NFAT5 contains non-canonical NLS and is transported into the nucleus by KPNB1. They also found that Exportin-T mediates the nuclear export of NFAT5 under hypotonicity. Furthermore, they also identified RUVBL2 as a chaperon that is essential for the nuclear export of NFAT5. From these findings the authors claimed that KPNB1 and RUVBL2 are key molecules responsible for the unconventional tonicity-regulated nucleocytoplasmic shuttling of NFAT5.

Since the mechanism of tonicity-dependent nucleocytoplasmic trafficking is still largely unknown, this study contains data of interest. The authors performed various experiments to identify the factors involved in the nucleocytoplasmic shuttling of NFAT5. The overall conclusion is supported by the data presented.
However, there are a few critical points that need to be examined in more detail in order to draw a convincing conclusion.

Comments for the author

One of the major concerns is the relevance of the findings to the regulation of endogenous NFAT5. Since the authors used various deletion mutants of NFAT5 (mainly 132-581) throughout the study, it is unclear whether the observed dynamics actually reflect those of endogenous NFAT5.
1. Does KPNB1, which was demonstrated to bind to FLAG-NFAT5(132-264) (Figure 3), also bind to full-length NFAT5? The authors can perform immunoprecipitation to test whether KPNB1 binds to endogenous NFAT5, as shown in Figure 5E. In addition, the effect of KPNB1 knockdown on the localization of endogenous NFAT5 should be investigated.
2. The authors showed that knockdown of XPOT (Figure 4) or RUVBL2 (Figure 5) mitigated hypotonicity-induced nuclear export of FLAG-NFAT5(132-581). Does knockdown of XPOT or RUVBL2 also suppress the nuclear export of endogenous NFAT5?
3. The relation between XPOT and RUVBL2 is not very clear. Is it possible that hypotonicity-induced nuclear export of RUVBL2 is mediated by XPOT? The authors can monitor the effect of XPOT knockdown on the nucleocytoplasmic localization of RUVBL2.

Minor comments:
1. Figure 3F: The values of Kds shown in the Figure 3F (11.4, 3.02, 4.7) are different from those in the text (1.2, 1.8, 1.8) .
2. Figure 3A: The efficiency of siRNA knockdown should be shown.

Reviewer 2

Advance summary and potential significance to field

The authors report an extensive investigation to identify the factors involved in the nucleocytoplasmic transport of the transcription factor NFAT5. In summary, the intracellular localization of NFAT5 varies according to the tonicity of the extracellular environment - in hypertonic conditions, NFAT5 is mostly localized in the nucleus and in hypotonic conditions, the protein is mostly localized to the cytoplasm. In this manuscript, the authors define the NLS of NFAT5 and NTRs involved in nuclear import and export of NFTAT5.
Previous work from the group (Tong et. al, 2006) identified residues 132-581 of NFAT5 as the minimal region required for nuclear import. In this manuscript, the authors show that the region

© 2022. Published by The Company of Biologists under the terms of the Creative Commons Attribution License (https://creativecommons.org/licenses/by/4.0/).

comprising residues 174-250 of NFAT5 is enough for nuclear import. The authors suggest that residues 199-216 that resemble a cNLS are necessary but not sufficient for nuclear import, and adjacent residues may be needed. The the authors showed that siRNA of KPNB1, but not of individual KPNA subtypes, abolished nuclear import of NFAT5. They also saw NFAT5-KPNB1 interactions via immunoprecipitation, pulldown binding assays with recombinant proteins, ITC and digitonin-permeabilized nuclear import assays. The experimental data for the nuclear import of NFAT5 is interesting, but siRNA analysis of KPNA subtypes is inconclusive and the low affinity NFAT5-KPNB1 binding is worrisome.

On the other hand, the authors suggest that XPOT is the major nuclear export receptor of NFAT5 and RUVBL2 is a chaperone required for the nuclear export of the protein. The suggestion of XPOT or XPO4 as potential nuclear exporters of NFTAT5 is supported by siRNA knockdown experiments only (Figure 4D). Therefore in the absence of in vitro studies showing direct binding of XPOT to NFAT5 these finding are a bit preliminary. The authors also performed several cellular experiments to investigate the role of RUVBL2 on the nuclear export of NFAT5 and the most conclusive data is summarized in the Figures 6B-D, 8A. Although these data clearly show that knockdown of RUVBL2 affects the nuclear export of NFTAT5, it is not clear if this is a direct or indirect effect. Perhaps Figure 7 and 6-A could be moved to supplementary information without significantly affect the story and could end up streamlining it.

Comments for the author

The following are specific comments:
1) Figure 1-B and 1-C: the nuclear translocation of the constructs FLAG-NFTA5132-581 and FLAG-NFTA5132-264-PEPCK is clearly visualized and very similar.
However, is it possible that the construct FLAG-NFAT5198-217 does not show similar nuclear localization due to an impediment from the FLAG-tag? The authors could have a positive control with a known cNLS sequence (eg SV40 T antigen cNLS) to show that the tag is not an impediment for NTR binding. The FLAG-tag in FLAG-NFAT5198-217 is close to the core 201RKR204 region that is clearly important nuclear import.
2) Figure 2: the discussion around the results from the EM images is very speculative and not conclusive. These findings could be mentioned briefly in the text and Figure 2 could be moved to supplementary.
3) Figure 3-D: siRNA experiments of the six KPNA subtypes are not conclusive. It is known that they all bind cNLSs and though there are unique cNLSs that bind one but not another, many cNLSs are expected to bind all KPNA subtypes. Therefore, no localization differences when any one of them is knocked down is not conclusive. A better experiment would be to inhibit all of them with the bimax or bimax2 peptide, which are potent inhibitors of all KPNAs.
4) Why are the siRNA-localization experiments performed with a small fragment of NFAT rather than full-length NFAT?
5) Figure 3-E: the binding assay is missing the inputs and the exposure for the image of the gel with lanes 7 and 8 is different. Ideally, all lanes should be in the same gel. In addition, this assay shows that residues 198-217 binds to KPNB1, which is contradictory with results of cellular localization assays involving this NFAT5 construct. In this case, there is only one N-terminal tag so the C-terminal tag in the cellular assays may be an impediment for the binding. The figure legend needs to clearly describe notations in the schematic.
For example, what do the dark blocks within the NFAT5 constructs refer to?
6) Figure 3-F: ITC experiments could have been performed in a lower concentration (1:10 or 1:20) of NFAT5 to guarantee that the first injections do not saturate KPNB1.
7) Most cargos bind importins with high affinity, in the tens of nM range.
The microM binding of NFAT5 to KPNB1 is unusual and worrisome. What about the same NFAT5 construct binding to KPNA proteins? Do they bind better or worse than KPNB1? Digitonin permeabilized nuclear import assays are in vitro assays performed using permeabilized cells, which reflect the ability of cargos to bind importins.
8) Figure 4: the panels A-C are related to nuclear import of NFAT5 and should be separated from panels D and E that are related to nuclear export.
Minor comments:
1) Introduction: In the 1st paragraph, the statement “Each of them recognizes a specific protein domain on protein cargos and ..” is inaccurate and confusing. In strict terms, a domain refers to an independently folded portion of a protein. Many NLSs are thus not domains as they are

© 2022. Published by The Company of Biologists under the terms of the Creative Commons Attribution License (https://creativecommons.org/licenses/by/4.0/).

linear targeting elements found in intrinsically disordered portions of cargo proteins. However there are also cargos that use folded domains to bind their importins.
Therefore, each importin recognizes specific linear nuclear targeting elements known as NLSs or specific folded domains in their cargos.
2) The last few sentences of the 1st paragraph is very outdated. The authors need to update their knowledge and rewrite accordingly.
“The best characterized protein domains being recognized by importins and exportins is the classical nuclear localization signals (cNLS) and canonical nuclear export signal (NES), respectively (Lange et al., 2007). Nevertheless nuclear import signals that do not conform to the cNLS, which are known as nonclassical nuclear localization signals (ncNLS), have also been identified (Soniat and Chook, 2015; Lee et al., 2006b).“
“best characterized protein domains” can be better replaced “The oldest characterized nuclear targeting elements are the classical nuclear localization signal (cNLS) and the classical nuclear export signal (NES).”
The concept of nonclassical nuclear localization signals is extremely outdated and superficial. It is unclear why the authors describe it this way since the reference they chose, Soniat and Chook, 2015, clearly describes 4 different types of very well characterized NLSs – cNLS, PY-NLS, IK-NLS and RS-NLS.

First revision

Author response to reviewers' comments

Response to reviewers

We thank the reviewers for their valuable comments that help us making the manuscript a better one. We apologize for the delay in response due to the pandemic. Here below please find our response to the reviewers.

Reviewer 1

1. Does KPNB1, which was demonstrated to bind to FLAG-NFAT5(132-264) (Figure 3), also bind to full-length NFAT5? The authors can perform immunoprecipitation to test whether KPNB1 binds to endogenous NFAT5, as shown in Figure 5E. In addition, the effect of KPNB1 knockdown on the localization of endogenous NFAT5 should be investigated.

Response: We conducted immuno-precipitation of Importin-beta1 and is able to detect NFAT5 signal in the immune-complex (Fig. 3C). Together with the evidence showing that KPNB1 knockdown altered subcellular localization of localization of NFAT5 under isotonic and hypertonic conditions (Fig. 1B), these data strongly suggested that importin beta directly interacts with NFAT5 and mediates its nuclear import.

2. The authors showed that knockdown of XPOT (Figure 4) or RUVBL2 (Figure 6) mitigated hypotonicity-induced nuclear export of FLAG-NFAT5(132-581). Does knockdown of XPOT or RUVBL2 also suppress the nuclear export of endogenous NFAT5?

Response: We have conducted siRNA knockdown of XPOT (Figure 4E) and RUVBL2 (Figure 6A) and found that depletion of either gene inhibits nuclear export of endogenous NFAT5 under hypotonicity.

3. The relation between XPOT and RUVBL2 is not very clear. Is it possible that hypotonicity-induced nuclear export of RUVBL2 is mediated by XPOT? The authors can monitor the effect of XPOT knockdown on the nucleocytoplasmic localization of RUVBL2.

Response: We have conducted siRNA knockdown XPOT and found that nuclear export of RUVBL2 under hypotonicity was inhibited (Fig. 7F). Accordingly, we have modified our model of NFAT5 nuclear export in the discussion

© 2022. Published by The Company of Biologists under the terms of the Creative Commons Attribution License (https://creativecommons.org/licenses/by/4.0/).

Minor comments:

1. Figure 3F: The values of Kds shown in the Figure 3F (11.4, 3.02, 4.7) are different from those in the text (1.2, 1.8, 1.8) .

Response: We apologize for oversight in not matching the ITC plots in Figure 3F with the text. Per advice from Reviewer #2, the ITC experiments were repeated to obtain data of better quality. New ITC data is presented in Figure 3G in this revised manuscript. The Kd values for the three NFAT5 constructs are (1.2, 1.2, 1.5), similar to the previous version.

2. Figure 3A: The efficiency of siRNA knockdown should be shown.

Response: The efficiency of siRNA knockdown was shown Supplementary Fig. 1B.

Reviewer 2 Advance Summary and Potential Significance to Field:
The authors report an extensive investigation to identify the factors involved in the nucleocytoplasmic transport of the transcription factor NFAT5. In summary, the intracellular localization of NFAT5 varies according to the tonicity of the extracellular environment - in hypertonic conditions, NFAT5 is mostly localized in the nucleus and in hypotonic conditions, the protein is mostly localized to the cytoplasm. In this manuscript, the authors define the NLS of NFAT5 and NTRs involved in nuclear import and export of NFTAT5. Previous work from the group (Tong et. al, 2006) identified residues 132-581 of NFAT5 as the minimal region required for nuclear import. In this manuscript, the authors show that the region comprising residues 174-250 of NFAT5 is enough for nuclear import. The authors suggest that residues 199-216 that resemble a cNLS are necessary but not sufficient for nuclear import, and adjacent residues may be needed. The authors showed that siRNA of KPNB1, but not of individual KPNA subtypes, abolished nuclear import of NFAT5. They also saw NFAT5-KPNB1 interactions via immunoprecipitation, pulldown binding assays with recombinant proteins, ITC and digitonin-permeabilized nuclear import assays. The experimental data for the nuclear import of NFAT5 is interesting, but siRNA analysis of KPNA subtypes is inconclusive and the low affinity NFAT5-KPNB1 binding is worrisome.

On the other hand, the authors suggest that XPOT is the major nuclear export receptor of NFAT5 and RUVBL2 is a chaperone required for the nuclear export of the protein. The suggestion of XPOT or XPO4 as potential nuclear exporters of NFTAT5 is supported by siRNA knockdown experiments only (Figure 4D). Therefore, in the absence of in vitro studies showing direct binding of XPOT to NFAT5, these finding are a bit preliminary. The authors also performed several cellular experiments to investigate the role of RUVBL2 on the nuclear export of NFAT5, and the most conclusive data is summarized in the Figures 6B-D, 8A. Although these data clearly show that knockdown of RUVBL2 affects the nuclear export of NFAT5, it is not clear if this is a direct or indirect effect. Perhaps Figure 7 and 6-A could be moved to supplementary information without significantly affect the story and could end up streamlining it.

Response: These figures showed very novel observations about the role of RUVBL2 in NFAT5 nuclear export. Although whether RUVBL2 exert a direct effect on NFAT5 export remains unknown, these data provided important clues for further research into RUVBL2 functions in this regard. Therefore, it will be helping and information to other researchers if they were shown in regular figure.

Reviewer 2 Comments for the Author:
The following are specific comments:

1) Figure 1-B and 1-C: the nuclear translocation of the constructs FLAG-NFTA5132-581 and FLAG-NFTA5132-264-PEPCK is clearly visualized and very similar. However, is it possible that the construct FLAG-NFAT5198-217 does not show similar nuclear localization due to an impediment from the FLAG-tag? The authors could have a positive control with a known cNLS sequence (eg SV40 T antigen cNLS) to show that the tag is not an impediment for NTR binding. The FLAG-tag in FLAG-NFAT5198-217 is close to the core 201RKR204 region that is clearly important nuclear import.

Response: We have generated FLAG-SV40cNLS-PEPCK and found that the construct entered the nucleus as expected (Fig 1C). Thus, FLAG epitope did not affect the function of the SV40NLS

© 2022. Published by The Company of Biologists under the terms of the Creative Commons Attribution License (https://creativecommons.org/licenses/by/4.0/).

despite it is located close the sequence.

2) Figure 2: the discussion around the results from the EM images is very speculative and not conclusive. These findings could be mentioned briefly in the text and Figure 2 could be moved to supplementary.

Response: This is the first attempt to probe for the localization of NFAT5 using a novel EM strategy, and lead to interesting results. We suggest to include them in the main text so the reader can make reference to them easily.

3) Figure 3-D: siRNA experiments of the six KPNA subtypes are not conclusive. It is known that they all bind cNLSs and though there are unique cNLSs that bind one but not another, many cNLSs are expected to bind all KPNA subtypes. Therefore, no localization differences when any one of them is knocked down is not conclusive. A better experiment would be to inhibit all of them with the bimax or bimax2 peptide, which are potent inhibitors of all KPNAs.

Response: We have conducted the bimax1 and bimax2 peptide experiment as suggested, and the data consistently suggested that NFAT5 nuclear import is not mediated by importin-alpha (Fig. 3E).

4) Why are the siRNA-localization experiments performed with a small fragment of NFAT rather than full-length NFAT?

Response: The reasons being that earlier studies from us have clearly already shown that 132- 581 a.a. residues of NFAT5 sufficiently direct tonicity-driven nucleocytoplasmic trafficking of it (1). In addition, we found that overexpression full-length NFAT5 is huge in size which post a challenge for efficient transfection. More importantly, we found that overexpression of full- length construct is toxic to cells. Therefore, we used the truncated from of the transcription factor for our analysis.

1. Tong EHY, Guo J-J, Huang A-L, Liu H, Hu C-D, Chung SSM, et al. Regulation of nucleocytoplasmic trafficking of transcription factor OREBP/TonEBP/NFAT5. The Journal of biological chemistry. 2006;281:23870–9.

5) Figure 3-E: the binding assay is missing the inputs and the exposure for the image of the gel with lanes 7 and 8 is different. Ideally, all lanes should be in the same gel. In addition, this assay shows that residues 198-217 binds to KPNB1, which is contradictory with results of cellular localization assays involving this NFAT5 construct. In this case, there is only one N- terminal tag, so the C-terminal tag in the cellular assays may be an impediment for the binding. The figure legend needs to clearly describe notations in the schematic. For example, what do the dark blocks within the NFAT5 constructs refer to?

Response: Firstly, the pull-down experiment has been repeated with all constructs A to G on the same gel. The new data is shown in Figure 3F. A gel showing all the inputs is included as Supplemental Figure 1D. Secondly, we agree with the reviewer that findings from the *in vitro* binding assay (i.e. a shorter cNLS-like region is able to interact with KPNB1) differ from results by in vitro reconstitution and cell assay (i.e. a longer region is required for nuclear import. Although the C-terminal tag (PEPCK) used in the cellular assays may be an impediment, the same tag did not impede the nuclear localization of SV40NLS (Figure 1C). Thus we believe a more likely reason is the longer region of NFAT5 may contain elements that enhance its binding to KPNB1, especially upon hypertonicity. Such enhancement may be essential for nuclear import in vivo but is not detected in in vitro binding assays. We have discussed such possibility in the Discussion section.

6) Figure 3-F: ITC experiments could have been performed in a lower concentration (1:10 or 1:20) of NFAT5 to guarantee that the first injections do not saturate KPNB1.

Response: We agree with the reviewer and have repeated the ITC experiments to improve data quality. We used purer protein samples and tried titrating with lower concentration. The new data is shown in Figure 3G. The issue of quick saturation after the first injection is noticeably improved for the shortest construct (189-216). For the longer constructs, the issue persisted even when lower concentration (1:20) was used. We suspect that the longer constructs may contain more than one

© 2022. Published by The Company of Biologists under the terms of the Creative Commons Attribution License (https://creativecommons.org/licenses/by/4.0/).

binding site for KPNB1, thus trickier to obtain the standard S-shaped curve. Overall, the Kd values obtained are similar for new vs. old data and among the three NFAT5 constructs (~1.2 uM).

7) Most cargos bind importins with high affinity, in the tens of nM range. The microM binding of NFAT5 to KPNB1 is unusual and worrisome. What about the same NFAT5construct binding to KPNA proteins? Do they bind better or worse than KPNB1?Digitonin permeabilized nuclear import assays are in vitro assays performed using permeabilized cells, which reflect the ability of cargos to bind importins.

Response: Indeed our digitonin permeabilized nuclear import assay clearly shown that KPNA protein is not involved in the binding. While KPNA is absolutely required for SV40-NLS mediated nuclear import, NFAT5-NLS-mediated nuclear import only requires KPNB1. Our new data using bimax1 and bimax2 further reinforced this finding. While NFAT5 binds to KPNB1 with lower affinity than most cargos, we would like to propose that this situation offers an opportunity for hypertonicity to strengthen this interaction and promote nuclear import of NFAT5. The exact molecular events triggered by hypertonicity are not well understood. It would be interesting to investigate how certain biophysical and biochemical factors like shear force, ion currents and polarity may affect NFAT5 and its interaction with KPNB1. We have included these statements in the Discussion section.

8) Figure 4: the panels A-C are related to nuclear import of NFAT5 and should be separated from panels D and E that are related to nuclear export.

Response: All figures are related to the study on the role of nuclear import/export receptors in NFAT5 nucleocytoplasmic trafficking. Therefore, we suggested that stay together.

Minor comments:

1) Introduction: In the 1st paragraph, the statement “Each of them recognizes a specific protein domain on protein cargos and ..” is inaccurate and confusing. In strict terms, a domain refers to an independently folded portion of a protein. Many NLSs are thus not domains as they are linear targeting elements found in intrinsically disordered portions of cargo proteins. However, there are also cargos that use folded domains to bind their importins. Therefore, each importin recognizes specific linear nuclear targeting elements known as NLSs or specific folded domains in their cargos.

Response: We have updated the writings.

2) The last few sentences of the 1st paragraph is very outdated. The authors need to update their knowledge and rewrite accordingly.

“The best characterized protein domains being recognized by importins and exportins is the classical nuclear localization signals (cNLS) and canonical nuclear export signal (NES), respectively (Lange et al., 2007). Nevertheless, nuclear import signals that do not conform to the cNLS, which are known as nonclassical nuclear localization signals (ncNLS), have also been identified (Soniat and Chook, 2015; Lee et al., 2006b).“

“best characterized protein domains” can be better replaced “The oldest characterized nuclear targeting elements are the classical nuclear localization signal (cNLS) and the classical nuclear export signal (NES).”

The concept of nonclassical nuclear localization signals is extremely outdated and superficial. It is unclear why the authors describe it this way since the reference they chose, Soniat and Chook, 2015, clearly describes 4 different types of very well characterized NLSs – cNLS, PY- NLS, IK-NLS and RS-NLS.

Response: We have updated writings.

© 2022. Published by The Company of Biologists under the terms of the Creative Commons Attribution License (https://creativecommons.org/licenses/by/4.0/).

Second decision letter

MS ID#: JOCES/2021/259280

MS TITLE: Unconventional tonicity-regulated nuclear trafficking of NFAT5 mediated by KPNB1, XPOT and RUVBL2

AUTHORS: Chris Y. Cheung, Ting-Ting Huang, Ning Chow, Shuqi Zhang, Yanxiang Zhao, Mary P. Chau, Ricky WC Chan, Catherine CL Wong, Daniela Boassa, Sebastien Phan, Mark H Ellisman, John Yates, SongXiao Xu, Zicheng Yu, Yajing Zhang, Rui Zhang, Ling Ling Ng, and Ben Chi Bun Ko
ARTICLE TYPE: Research Article

I am happy to tell you that your manuscript has been accepted for publication in Journal of Cell Science, pending standard ethics checks.

Reviewer 1

Advance summary and potential significance to field

/

Comments for the author

The authors adequately responded to my comments.
p16 To further elucidate if RUVBL2 nuclear export requires
p20, monomeric

Reviewer 2

Advance summary and potential significance to field

The authors report an extensive investigation to identify the factors involved in the nucleocytoplasmic transport of the transcription factor NFAT5. In summary the intracellular localization of NFAT5 varies according to the tonicity of the extracellular environment - in hypertonic conditions, NFAT5 is mostly localized in the nucleus and in hypotonic conditions, the protein is mostly localized to the cytoplasm. In this manuscript, the authors define the NLS of NFAT5 and NTRs involved in nuclear import and export of NFTAT5.

Comments for the author

The authors performed new experiments that support their findings that KAPB1 alone can transport NFAT5 to the nucleus. They also performed new experiments that support the hypothesis that XPOT is an export receptor for NFAT5 and its binding is mediated by RUVBL2. Therefore, their findings suggest a new nucleocytoplasmic pathway for the transcription factor NFAT5, which could lead to interesting new discoveries in the field, therefore, it should be accepted for publication.

© 2022. Published by The Company of Biologists under the terms of the Creative Commons Attribution License (https://creativecommons.org/licenses/by/4.0/).
